# Excited-State-Altering Ratiometric Fluorescent Probes for the Response of *β*-Galactosidase in Senescent Cells

**DOI:** 10.3390/molecules30061221

**Published:** 2025-03-08

**Authors:** Ya-Nan Han, Lei Dong, Lu-Lu Sun, Wen-Jia Li, Jianjing Xie, Congyu Li, Shuhui Ren, Zhan Zhang, Hai-Hao Han, Zhong Zhang

**Affiliations:** 1 School of Life Science and Technology, Shandong Second Medical University, Weifang 261053, China; hanyanan0911@163.com (Y.-N.H.); xiejianjing955@163.com (J.X.); licongyua@163.com (C.L.); rensh1012@163.com (S.R.); 2 School of Pharmacy, Shandong Second Medical University, Weifang 261053, China; 3 Shandong Laboratory of Yantai Drug Discovery, Bohai Rim Advanced Research Institute for Drug Discovery, Yantai 264117, China; llsun@baridd.ac.cn (L.-L.S.); liwenjia@simm.ac.cn (W.-J.L.); 4 Molecular Imaging Center, Stake Key Laboratory of Chemical Biology, Shanghai Institute of Materia Medica, Chinese Academy of Sciences, Shanghai 201203, China; 5 School of Chemistry and Chemical Engineering, Yangzhou University, Yangzhou 225002, China; zhanzhang@yzu.edu.cn

**Keywords:** *β*-galactosidase, excitation variation, fluorescent imaging, cell senescence

## Abstract

*β*-galactosidase (*β*-Gal) has emerged as a pivotal biomarker for the comprehensive investigation of diseases associated with cellular senescence. The development of a fluorescent sensor is of considerable importance for precisely detecting the activity and spatial distribution of *β*-Gal. In this study, we developed two excited-state-altering responsive fluorescent sensors (**TF1** and **TF2**) for ratiometric detection of *β*-Gal. Two TCF dyes, composed of tricyanofuran (TCF) and naphthol units, feature electron “pull–push” systems and are quenched fluorescence by *β*-Gal. Upon *β*-Gal hydrolysis, a significant ratiometric shift in absorption from ca. 475 nm to 630 nm is observed, accompanied by the emergence of a fluorescence signal at ca. 660 nm. The enzyme-responsive optical red-shifts are attributed to the excited-state transition from intramolecular charge transfer (ICT) state to local excited (LE) state, which was confirmed by density functional theory (DFT) calculations. Both fluorescent sensors display exceptional sensitivity and selectivity for the response of *β*-Gal in PBS solution and are capable of tracking *β*-Gal within senescent A549 cells. This study introduces a framework for developing multimodal optical probes by systematically modulating excited-state properties, demonstrating their utility in senescence studies, diagnostic assay design, and therapeutic assessment.

## 1. Introduction

Cell senescence is a pivotal event in the senescent process of organisms and holds significant importance for the investigation of disease onset and therapeutic strategies [1,2,3,4]. *β*-Galactosidase (*β*-Gal), a member of the glycoside hydrolase enzymes, has emerged as a crucial biomarker that is overexpressed in senescent cells [5,6,7]. The sensitive and precise detection of intracellular *β*-Gal levels is instrumental in tracking senescent cells and delving deeper into the intricacies of cellular senescence [8,9]. Traditional analytical methods usually have a more limited ability to dynamically visualize *β*-Gal activity within living cells. Fluorescence analysis and imaging offer a suite of advantages, including high sensitivity, accurate spatiotemporal resolution, and non-invasive monitoring [10,11,12,13]. Therefore, the development of novel fluorescent probes, which can sensitively and selectively respond to *β*-Gal, is paramount for the advancement of fluorescence-based assays in the detection and sensing of senescent cells.

At present, researchers are increasingly concentrating their efforts on the development of fluorescent probes for the sensitive detection of *β*-Gal [11,14,15,16,17]. The majority of probes operate by emitting a fluorescent signal in response to *β*-Gal’s catalytic hydrolysis of the galactose moiety [18,19]. The reported fluorescent sensors can be simply categorized into three types: (i) The probes in situ label on the *β*-Gal and generate a fluorescence signal followed by the hydrolysis of galactosides [20,21,22,23]. (ii) The sensors initially exhibit quenched fluorescence by conjugating with galactosides. After enzyme hydrolysis, an enhanced fluorescence can be observed as the original excited state of fluorophores is restored. However, the “turn-on” probes usually confront the interference from a biological background, leading to the low signal-to-noise ratio in vivo imaging [24,25,26,27]. (iii) Ratiometric probes display a shifted absorption and emission wavelength after reacting with *β*-Gal, which provides more accurate analysis by monitoring the *β*-Gal induced intensity variation between the original and shifted wavelength [28,29]. Consequently, the development of novel ratiometric probes could significantly enhance the analysis and imaging of *β*-Gal. Despite a great deal of potential, such probes are still rarely reported.

Tricyanofuran (TCF) serves as a prominent electron-deficient scaffold, widely employed in designing fluorophores with pronounced intramolecular charge transfer (ICT) characteristics. At present, TCF-based fluorescent probes are frequently reported for intracellular biomarker sensing [30,31,32]. Current TCF-derived optical sensors feature extended π-conjugation systems, which were formed by linking the electron-deficient TCF core with electron-donating moieties through conjugated bridges such as polyene chains or aromatic heterocycles (e.g., furan or thiophene). These “D-π-A” dyes typically demonstrate broad absorption spectra (500–600 nm) accompanied by NIR emission beyond 700 nm, exhibiting large stokes-shifts consistent with conventional ICT mechanisms. However, some TCF probes adopt simplified structures comprising TCF directly coupled with phenolic derivatives. Surprisingly, these compact π-conjugation systems display contradictory photophysical behavior with sharp absorption bands at longer wavelengths and minimal stokes-shifts. These anomalous characteristics deviate from typical ICT-mediated optical patterns. Thus, understanding the potential reasons for the spectral changes of dyes is of great significance for accurate *β*-Gal sensing and designing other kinds of probes.

**Figure 1:**
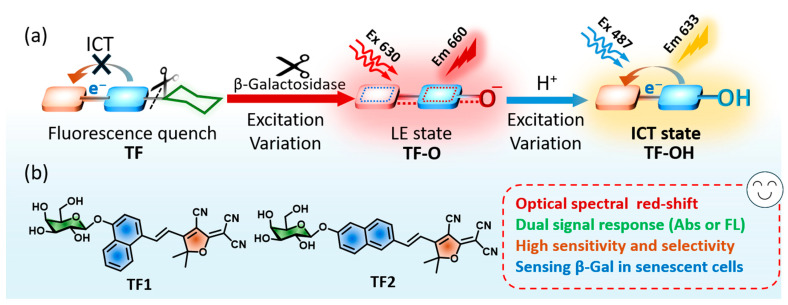
(**a**) *β*-Gal-responded fluorescent probes provide ratiometric optical signal by excited-state altering from ICT to LE states. (**b**) Structures of TCF-based fluorescent probes (**TF1** and **TF2**).

In this study, we develop two fluorescent probes to ratiometrically track *β*-galactosidase through excited-state variation from intramolecular charge transfer (ICT) state to local excitation (LE) state (Figure 1). Tricyanofuran (TCF) and naphthol derivative are conjugated with the electronic “pull–push” structures; they also introduce galactosides to form probes **TF1** and **TF2** (Figure 1b). The probes possess low absorption at 475 nm and quenched fluorescence at 613 nm, and their optical properties are dominated by the ICT state. When *β*-Gal hydrolyzes the probes, the optical spectra of residues (**TF-O**) are mainly influenced by the LE state, exhibiting both red-shifted and enhanced absorption and emission located at ca. 630 nm and 660 nm, respectively. Interestingly, the ICT effect can be restored when residues change type, from naphthoxide anion (**TF-O**) to neutral naphthol (**TF-OH**), followed by the blue-shifted absorption at 487 nm and fluorescence at 633 nm (Figure 1a). The density functional theory (DFT) calculation certifies the excited-state variation in different situations of probes. Two probes are allowed to rapidly detect *β*-Gal with high sensitivity and selectivity. Thanks to the ratiometric optical properties, the limit of detection for **TF1** is optimized to 0.003 U mL^−1^ for fluorescence and 0.0002 U mL^−1^ for absorption. Lastly, **TF1** and **TF2** can persistently monitor the endogenous *β*-Gal in senescent A549 cells for 24 h.

## 2. Results and Discussion

### 2.1. Structure and Photophysical Properties

We noticed that, after responding to analytes, some TCF-based probes exhibited optical red-shifts that did not seem to fit the signature of ICT-dominant fluorophores. To use the ratiometric optical variation and explore the underlying reasons for these changes, we continued to select TCF as the electron acceptor, conjugated with electron-rich naphthol units to construct the ICT architecture. Galactoside was modified on the hydroxy moiety to interrupt the electron “pull–push” system and quench the fluorescence. TCF can be one-step synthesized via malononitrile and 3-hydroxy-3-methylbutan-2-one at a high reaction yield [33]. Then, 4-Hydroxy-1-naphthaldehyde or 6-hydroxy-2-naphthaldehyde was conjugated with TCF through Knoevenagel condensation to form the fluorophores **TF1-OH** or **TF2-OH**, respectively. The galactosyl moiety was introduced into fluorophore through glycosylation reaction, and then underwent solvolysis in a basic environment to afford the targeting probes **TF1** and **TF2** (Schemes S1 and S2). The relevant characteristics, including ^1^H NMR, ^13^C NMR and high-resolution mass spectrometry (HRMS), were shown in the Supporting Information.

With two probes in hand, we initially investigated the photophysical variations of **TF1** and **TF2** for the response of *β*-Gal. A yellow solution of **TF1** or **TF2** displayed wide absorption band at 475 m (*ε* = 1.14 × 10^4^ L mol^−1^ cm^−1^) and 460 nm (*ε* = 1.19 × 10^4^ L mol^−1^ cm^−1^) and feeble fluorescence at 613 nm and 604 nm in PBS buffer (pH 7.4, 1 mM) mixed with 10% DMSO, respectively. Surprisingly, the sample color of **TF1** was distinctly changed to blue upon addition of *β*-Gal (4 U mL^−1^). The residue of **TF1** exhibited a red-shifted absorption at 632 nm (*ε* = 8.01 × 10^4^ L mol^−1^ cm^−1^) and fluorescence at 661 nm (*Φ*_F_ = 1.25%). The residue of **TF2** was monitored to discern any similar variations of the optical property, i.e., that the wavelength of absorption and fluorescence maxima were located at 631 nm (*ε* = 5.2 × 10^4^ L mol^−1^ cm^−1^) and 659 nm (*Φ*_F_ = 1.03%), respectively (Figure 1a,b, Table S1). In comparison to the large stokes-shifts (ca. 140 nm) of **TF**s (**TF1** and **TF2**), the residues possess shrunken stokes-shifts of less than 30 nm (Table S1). It is well known that ICT-based fluorescent probes generally exhibit fluorescence enhancement and slight absorption variation with large stokes-shifts, rather than drastic red-shifts and increased absorption with small stokes-shifts [34,35,36].

**Figure 2:**
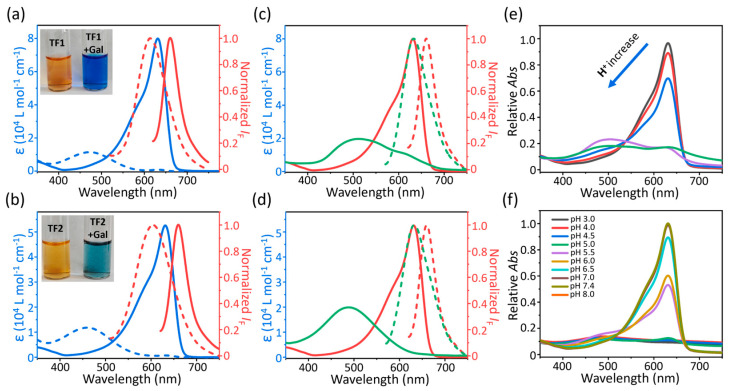
Molar extinction coefficient (*ε*, dash line) and normalized fluorescence (line) of (**a**) **TF1** and (**b**) **TF2** (10 μM) incubated without (blue) or with *β*-Gal (red, 4 U mL^−1^) at 37 °C for 1 h. Inset: Sample photos of probe alone (left) and probe incubated with *β*-Gal (right, 4 U mL^−1^). Molar extinction coeffcient (*ε*, solid line) and normalized fluorescence (dashed line) of residues from *β*-Gal-cut (**c**) **TF1** and (**d**) **TF2** (10 μM) after adding with (green, **TF-OH**) or without (red, **TF-O**) trifluoroacetic acid (50 μL). (**e**) Relative absorption of samples of *β*-Gal-cut **TF1** with addition of trifluoroacetic acid (50 μL, interval 10 μL). (**f**) Relative absorption variation of **TF1-OH** in 10% DMSO-mixed PBS buffer with different pH values (3–8).

We speculated that excited-state variation or formation of J-aggregates might induce the unusual optical properties of residues. Based on the reported hydrolysis mechanism of *β*-Gal, the glycoside bond connecting fluorophore and galactoside was cut off, immediately generating two types of residues: naphthol (**TF-OH**) or naphthoxide anion (**TF-O**) [18]. Hence, we added trifluoroacetic acid (TFA, 50 μL) into the *β*-Gal-incubated probe solution, and then measured the photophysical properties. While adding H^+^ into the *β*-Gal-cut **TF1** sample, the solution color rapidly transferred from blue to light purple (Figure S1). Compared with residues, the H^+^-added sample exhibited a blue-shifted, wide and weak absorption at 487 nm (*ε* = 2.02 × 10^4^ L mol^−1^ cm^−1^), followed by a blue-shifted but increased fluorescence at 633 nm (*Φ*_F_ = 1.79%). The optical variation of **TF2** residues was similar to **TF1** after adding TFA (Figure 2c,d, Table S1). Subsequently, we observed a ratiometric absorption blue-shift of **TF1** residues from 632 nm to 487 nm upon general addition of TFA (0–50 μL, interval 10 μL, Figure 2e). The absorptions of **TF1-OH** in the aqueous environment with different pH values (3–8) were recorded. A red-shifted and increased absorption band occurred at 632 nm, following the pH varying to neutral, which indicated that the conjugated base of **TF1-OH** was generated in the more alkaline environments (Figure 2f). In addition, mass spectrometry observed the mass peak of residues at 352.11 (Figure S2) but could not find the corresponding mass peaks of **TF**s. These results sufficiently indicated that (i) the attractive optical spectra of residues were not triggered by intermolecular J-aggregation; (ii) the *β*-Gal hydrolyzed residues are probably the naphthoxide anion type, named **TF-O**, rather than naphthol (**TF-OH**, Scheme S3).

### 2.2. DFT Caculation of Electron Distribution

Excluding J-aggregation, we hypothesized that the ICT effect might not be the main factor to influence the photophysical properties of **TF-O**s. Therefore, the highest occupied molecular orbitals (HOMOs), the lowest unoccupied molecular orbitals (LUMOs), and the excited energy of the S_1_ state for **TF**s, **TF-O**s, and **TF-OH**s were calculated using the density functional theory (DFT) method at the B3LYP/6-31G(d,p) level of theory in the gas phase (Tables S2–S4). Both probes (TFs) and their corresponding naphthols (**TF-OH**s) showed that the electrons on the HOMO levels were distributed on the whole molecules, whereas LUMOs were partial to TCF units. The distinct electron transfer suggested that the ICT effect probably contributed to the optical properties of **TF**s and **TF-OH**s (Figure S3, left and middle). However, the HOMOs and LUMOs were located on the entire backbone of **TF-O**s, instead of the electron movements from donor to acceptor (Figure S3, right).

**Figure 3:**
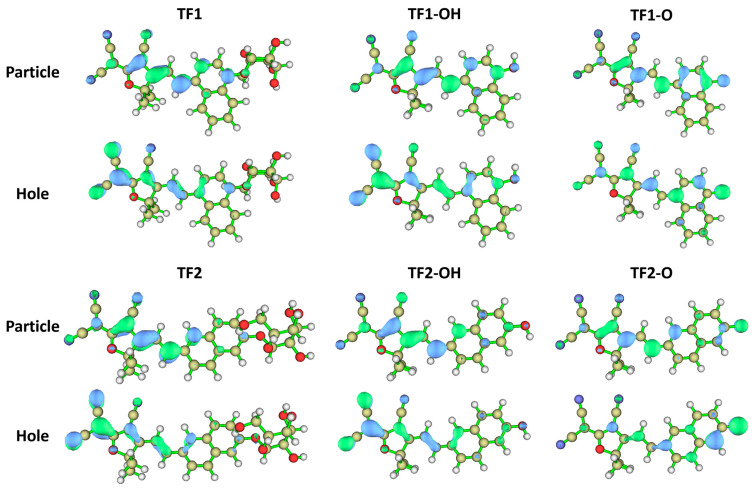
Calculated natural transition orbitals (NTOs) of **TF1**, **TF2**, **TF1-OH**, **TF2-OH**, **TF1-O** and **TF2-O** using density functional theory at the PBE1PBE/6-31G* level of theory in the gas phase.

To further explore the electronic migration in the excited state, we calculated the natural transition orbitals (NTOs) within the S_1_ excited state by [37,38,39,40,41]. From the NTOs analysis, we observed that the holes of **TFs** and **TF-OH**s were attracted on the electron-deficient malononitrile, whereas the electrons in the excited-state were mainly distributed on the π-conjugated backbone containing the furan ring of TCF, double bond, and phenyl groups. The clear electronic migration in the excited state indicated that both **TFs** and **TF-OH**s maintain the ICT-controlled photophysical characters, including broad absorption and emission bands with large stokes-shifts. In contrast, the hole and particle distributions of **TF-O**s were significantly overlapped on the molecular scaffold in the excited state, rather than being concentrated on the electronic acceptor of TCF (Figure 3). The NTOs assay could sufficiently demonstrate the electronic distribution difference between **TF**s, **TF-Oh**s, and **TF-O**s in the excited state, thereby influencing their respective optical spectra. The diagrams of electron-hole distributions in the S_1_ excited state were in agreement with NTO analysis, indicating that the excited-state of **TF**s and **TF-OH**s was different with **TF-O**s (Figure S4). Considering the unique optical spectra with narrow absorption and emission bands, increased molar extinction coefficient, and reduced stokes-shifts (less than 30 nm) [42,43], we speculated that the local excited (LE) state probably dominated the photophysical properties of **TF-O**s, which might undergo a fast structural transformation between naphthoxide anions and naphthoquinone, as is reported with TCF-based cyanine dye (Scheme S4) [44].

### 2.3. Detection of β-Gal in Solution

**Figure 4:**
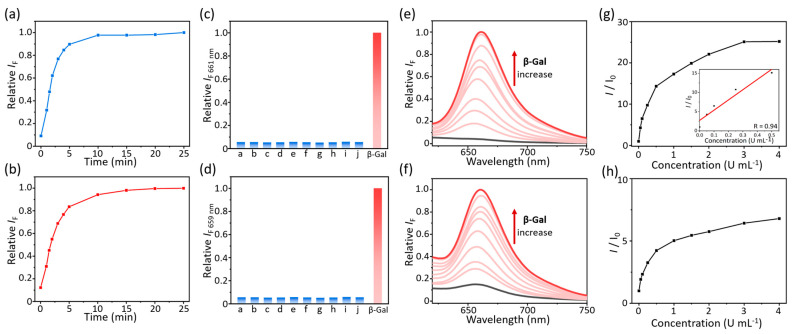
Plotting the relative fluorescence changes of (**a**) **TF1** and (**b**) **TF2** (10 μM) incubated with *β*-Gal (4 U mL^−1^) as a function of time. Relative fluorescence changes of (**c**) **TF1** and (**d**) **TF2** (10 μM) incubated with *β*-Gal (4 U mL^−1^) and other analytes for 25 min. [a: blank, b: H_2_O_2_, c: NaClO, d: Hcy, e: Cys, f: GSH, (a–f, 200 μM), g: DNA, h: BSA, i: cellose (g–i, 200 μg mL^−1^), j: *β*-glucosidase (4 U mL^−1^)]. Fluorescence spectra of (**e**) **TF1** and (**f**) **TF2** (10 μM) incubated with different concentrations of *β*-Gal (0–4 U mL^−1^). Plotting the *I*/*I*_0_ of (**g**) **TF1** and (**h**) **TF2** (10 μM) incubated with different concentrations of *β*-Gal (0–4 U mL^−1^), inset: fitted *I*/*I*_0_ curve of **TF1** incubated with a low concentration of *β*-Gal (0–0.5 U mL^−1^). *I* and *I*_0_ are the fluorescence intensity of probes in the presence and absence of *β*-Gal. All fluorescence spectra were excited at 570 nm and measured at 37 °C in PBS (pH 7.4) mixed with 10% DMSO.

By utilizing ratiometric absorption and activatable fluorescence at ca. 660 nm, we evaluated the detection capability of **TF1** and **TF2** targeting at *β*-Gal. The absorption and fluorescence changes of TFs were recorded in the presence of *β*-Gal (4 U mL^−1^) incubated for different lengths of time. When the time was increased, a ratiometic absorption variation was observed from 475 nm to 632 nm, followed by a time-dependent fluorescence enhancement at 661 nm (λ_ex_ = 570 nm, Figure S5). Similar optical spectrum changes in **TF2** were measured while incubated *β*-Gal within 25 min (Figure S6). For both **TF1** and **TF2**, the fluorescence was dramatically enhanced until a plateau was reached after the enzyme had been incubated for over 10 min, indicating that the two probes could rapidly detect the *β*-Gal as well as generate the color or fluorescence difference in the solution (Figure 4a,b and Figure S7). Then, the absorption and fluorescence spectra of probes were measured in the presence of different analytes, including H_2_O_2_, NaClO, homocysteine (Hcy), cysteine (Cys), glutathione (GSH), deoxyribonucleic acid (DNA), Bull Serum Albumin (BSA), cellose, *β*-glucosidase, and *β*-Gal. Besides the glaring emission of *β*-Gal, other analytes cannot “turn-on” the fluorescence of **TF1** or **TF2** at ca. 660 nm. The glycosidic bonds could only be hydrolyzed by *β*-Gal, indicating that the probes have satisfied selectivity for the enzyme (Figure 4c,d, Figures S8a and S9a). The absorption spectra further demonstrated the single-response ability targeting *β*-Gal in solution (Figures S8b,c and S9b,c).

Next, the absorption and fluorescence changes of **TF1** and **TF2** were respectively evaluated upon the concentration increase (0–4 U mL^−1^) of incubated *β*-Gal at 37 °C for 25 min. We observed a concentration-dependent fluorescence enhancement of two probes for *β*-Gal at ca. 660 nm (λ_ex_ = 570 nm), followed by a gradual red-shift and increase in absorption of **TF**s (Figure 4e,f and Figure S10a,b). When the fluorescence reached a plateau, the intensity of **TF1** had increased by about 25-fold compared to the initial emission, which was higher than that of **TF2** (Figure 4g,h). A good linearity was fitted by plotting the fluorescence enhancement of **TF1** as a function of increasing *β*-Gal from 0 to 0.5 U mL^−1^, and a limit of detection (LOD) was determined as 0.003 U mL^−1^ (*3σ/k*, Figure 4g inset). Due to the ratiometric response, the line was fitted by plotting the absorption ratio between 632 nm and 475 nm, resulting in a more sensitive LOD as 0.0002 mL^−1^ (Figure S10c). Therefore, **TF1** possesses a fascinating application potential as it can not only respond to *β*-Gal through fluorescence sensing, but also determine the concentration of *β*-Gal in solution samples by measuring absorption variation or using colorimetric methods.

### 2.4. Sensing β-Gal in Senescent Cells

**Figure 5:**
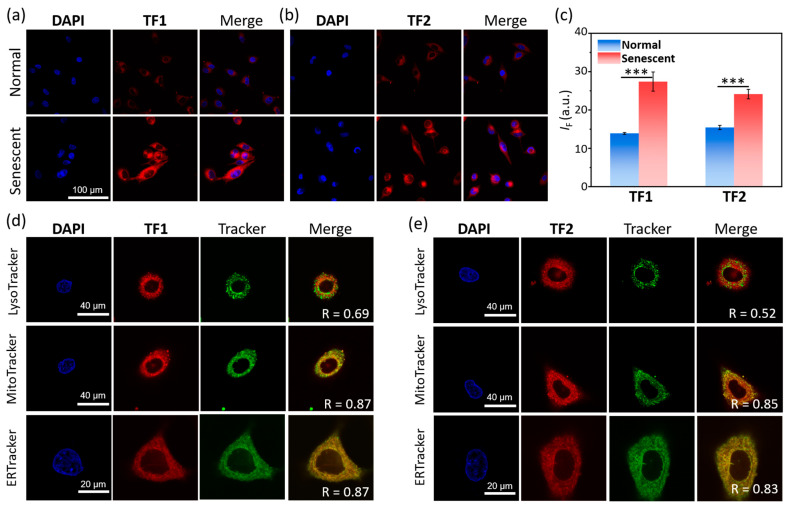
Fluorescence imaging of normal and senescent A549 cells incubated with (**a**) **TF1** and (**b**) **TF2** (10 μM) for 4 h. (**c**) Fluorescence quantification of normal (blue) and senescent (red) A549 cells incubated with **TF1** and **TF2** (10 μM) for 4 h. Fluorescence co-localization of (**d**) **TF1** and (**e**) **TF2** (10 μM) with LysoTracker (a lysosome tracker, 75 nM), MitoTracker (a mitochondria tracker, 75 nM)), and ERTracker (an endoplasmic reticulum tracker, 75 nM). Excitation and emission wavelength of **TF**s were 640 nm and 650–700 nm, respectively. Error bars represent the S.D. *** *p* < 0.001; *n* = 3.

Based on the outstanding sensitivity and selectivity of **TF1** and **TF2** for *β*-Gal in solution, we turned our view to utilizing two probes for fluorescence imaging of *β*-Gal in cells. Due to the overexpression of *β*-Gal during cell senescence [4], we examined the fluorescence sensing of *β*-Gal in senescent and normal (control) A549 cells (lung cancer). We initially evaluated the cytotoxicity of two probes at a variation of concentrations after incubating with normal and senescent A549 cells for 24 h. The results indicated that **TF1** and **TF2** (0–10 μM) exhibited low cytotoxicity for cell proliferation of both normal and senescent A549 cells (Figure S11). Then, **TF1** and **TF2** were respectively incubated with senescent and normal A549 cells for 4 h, and the fluorescence signal was collected (650–700 nm) under excitation at 640 nm. We determined that the fluorescence intensity of **TF1** in senescent cells was twice as high as that in normal cells, demonstrating that the overexpressed *β*-Gal was present in the cell senescence progress (Figure 5a,c). **TF2** could also sensitively monitor the concentration difference of *β*-Gal between senescent and control groups (Figure 5b,c). A concentration-dependent fluorescence enhancement of **TF1** and **TF2** was observed in senescent A549 cells (Figures S12 and S13). In addition, the fluorescence intensity gradually kept brightening until the plateau was attained after ~6 h of incubation (Figures S14 and S15). It is worth noting that **TF1** and **TF2** still provided an unattenuated fluorescence signal during 24 h, revealing that two probes allowed a long-period of fluorescence tracking that targeted *β*-Gal. Finally, we carried out the co-staining assay to explore the subcellular localization of **TF**s. After comparing the fluorescence overlap between **TF**s and commercial subcellular trackers, we supposed that the two probes exhibited similar subcellular localization, distributing in both the mitochondria and endoplasmic reticulum, rather than the lysosome (Figure 5d,e, Figures S16 and S17).

## 3. Materials and Methods

### 3.1. Synthesis

All reagents for synthesis commercially available were used without further purification (Anhui Senrise Technologies Co., Ltd., Hefei, China). Compounds **TF1** and **TF2** were synthesized according to the same protocols. The glycosylated compound (**TF-GalAc**) was sufficiently dissolved in MeOH (30 mL) mixed with tetrahydrofuran (THF, 2–3 mL). Sodium methoxide (MeONa) was subsequently added to the solution, which was then stirred at room temperature for 4 h. Thin-layer chromatography (TLC) was employed to monitor the reaction until the starting materials had completely disappeared. The reaction was neutralized with a 2 M HCl aqueous solution, evaporated to dryness, and purified using column chromatography (CH_2_Cl_2_:MeOH = 15:1, *v*/*v*) to obtain the targeting compound **TF**s.

Starting from compound **TF1-GalAc** (170 mg, 0.249 mmol, 1 eq.) and MeONa (81 mg, 1.49 mmol, 6 eq.), compound **TF1** was synthesized as an amorphous dark-red solid (76 mg, 76%). ^1^H NMR (400 MHz, DMSO-*d*_6_): *δ* (ppm) 8.85 (d, *J* = 16.0 Hz, 1H), 8.46 (d, *J* = 8.3 Hz, 1H), 8.38 (d, *J* = 8.4 Hz, 1H), 8.25 (d, *J* = 8.6 Hz, 1H), 7.76 (t, *J* = 7.7 Hz, 1H), 7.65 (t, *J* = 7.7 Hz, 1H), 7.35 (s, 1H), 7.34 (d, *J* = 25.8 Hz, 1H), 5.43 (d, *J* = 5.3 Hz, 1H), 5.17 (d, *J* = 7.7 Hz, 1H), 4.96 (d, *J* = 5.7 Hz, 1H), 4.70 (t, *J* = 5.4 Hz, 1H), 4.61 (d, *J* = 4.6 Hz, 1H), 3.85–3.72 (m, 3H), 3.59–3.51 (m, 3H), 1.82 (s, 6H). ^13^C NMR (101 MHz, DMSO-*d*_6_): *δ* (ppm) 25.0, 53.8, 60.5, 68.2, 70.3, 73.1, 76.0, 96.7, 99.4, 101.1, 109.1, 112.0, 112.9, 115.0, 122.3, 123.2, 124.1, 125.1, 126.2, 128.6, 128.8, 131.6, 131.7, 132.4, 143.0, 157.1, 175.8, 177.5. HR-ESI-MS *m*/*z*: calcd. for C_28_H_25_N_3_O_7_Na [M+Na]^+^ 538.1590 found 538.1594.

Starting from compound **TF2-GalAc** (160 mg, 0.234 mmol, 1 eq.) and MeONa (76 mg, 1.40 mmol, 6 eq.) compound **TF2** was synthesized as an amorphous dark-red solid (54 mg, 65%).^1^H NMR (600 MHz, DMSO-*d*_6_): *δ* (ppm) 8.86 (d, *J* = 16.0 Hz, 1H), 8.46 (d, *J* = 8.5 Hz, 1H), 8.39 (d, *J* = 8.5 Hz, 1H), 8.25 (d, *J* = 8.8 Hz, 1H), 7.76 (t, *J* = 7.0 Hz, 1zH), 7.67–7.64 (m, 1H), 7.37–7.31 (m, 2H), 5.18 (d, *J* = 7.7 Hz, 2H), 3.82 (t, *J* = 8.6 Hz, 1H), 3.77 (d, *J* = 3.0 Hz, 1H), 3.74 (t, *J* = 6.3 Hz, 1H), 3.61–3.49 (m, 3H), 1.82 (s, 6H). ^13^C NMR (150 MHz, DMSO-*d*_6_): *δ* (ppm) 25.4, 54.2, 60.9, 68.7, 70.8, 73.6, 76.4, 97.1, 99.9, 101.5, 109.5, 112.5, 113.3, 115.4, 122.8, 123.7, 124.6, 125.6, 126.6, 129.1, 129.2, 132.8, 143.5, 157.6, 176.3, 178.0. HR-ESI-MS *m*/*z*: calcd. for C_28_H_25_N_3_O_7_Na [M+Na]^+^ 538.1590 found 538.1595.

### 3.2. UV-Vis and Fluorescence Spectroscopy

UV-visible spectra were analyzed using a Hitachi UV-2550 spectrophotometer (Hitachi High-Technologies Co., Tokyo, Japan). All spectra were corrected for background intensities by subtracting the spectra of pure solvent measured under identical conditions. The fluorescence measurements were carried out at room temperature using a Hitachi F-4600 spectrophotometer (Hitachi High-Technologies Co., Tokyo, Japan, slit width 5–10 nm, 700 V). The fluorescence of residue was excited at 570 nm and the emission signal was collected at 590–750 nm. The relative absorption (*Abs*) and relative emission intensity (*I*_F_) were normalized by setting the maximum values of the spectra to 1, with all other values scaled proportionally within the [0,1] range.

### 3.3. β-Gal Hydrolysis in Solution

First, 10 μL DMSO solution of probe (**TF1** or **TF2**, 2 mM) and 10 μL PBS solution of *β*-Gal (800 U mL^−1^) were added to 1980 μL PBS buffer (pH 7.4, 1 mM) mixed with 10% DMSO to prepare a testing sample (2 mL) with concentrations of probe and *β*-Gal as 10 μM and 4 U mL^−1^, respectively. Then, the resulting sample was placed at 37 °C for 1 h before measuring the absorption and fluorescence spectra.

### 3.4. Cell Culture

A549 cells (CCL-185^TM^) were obtained from the American Type Culture Collection (ATCC, Alexandria, MN, USA), which were cultured in RPMI-1640 supplemented with 10% FBS, 1% penicillin/streptomycin in a humidified incubator at 37 °C and 5% CO_2_. To obtain drug-induced senescent cells (SnCs), A549 cells were stimulated with mitomycin C (MitoC, 0.5 µM) twice for 2 days.

### 3.5. Cellular Response of Probes to β-Gal

To study the response of the **TF1** and **TF2** probes, senescent and normal A549 cells were seeded onto 24-well plates and incubated with a **TF1** or **TF2** probe (10 μM) for 4 h. Then, cells were fixed with 4% PFA and washed with phosphate buffered saline (PBS) three times before imaging. A laser scanning confocal microscopy (Olympus-FV3000, Olympus Corporation, Tokyo, Japan) was used to obtain an image at E_x_ = 640 nm and E_m_ = 650–700 nm. **TF1**: Confocal gain was set to 1-fold. PMT voltage was 550 V and laser transmissivity was 3%. **TF2**: Confocal gain was set to 1-fold. PMT voltage was 540 V and laser transmissivity was 3%. All images were acquired with 1.612 s intervals between frames for two frames. Frame size was 1024 × 1024.

### 3.6. Fluorescence Co-Localization Experiments

To explore the co-localization with probes, senescent A549 cells were stained with 10 μM **TF1** or **TF2** for 4 h, followed by 75 nM MitoTracker or LysoTracker for 1 h. Cells were fixed with 4% PFA and washed with phosphate buffered saline (PBS) three times before imaging. A laser scanning confocal microscopy (Olympus-FV3000, Olympus Corporation, Tokyo, Japan) was used to obtain images (**TF1** or **TF2**, probes: E_x_ = 640 nm and E_m_ = 650–700 nm, Tracker: E_x_ = 488 nm and E_m_ = 500–530 nm). **TF1**: Confocal gain was set to 1-fold. PMT voltage was 580 V and laser transmissivity was 7%. **TF2**: Confocal gain was set to 1-fold. PMT voltage was 560 V and laser transmissivity was 3%. MitoTracker: Confocal gain was set to 1-fold. PMT voltage was 510 V and laser transmissivity was 7%. LysoTracker: Confocal gain was set to 1-fold. PMT voltage was 480 V and laser transmissivity was 0.5%. All images were acquired with 1.612 s intervals between frames for two frames. Frame size was 1024 × 1024.

## 4. Conclusions

In summary, we successfully developed two novel fluorescent probes, **TF1** and **TF2**, that can ratiometrically track *β*-galactosidase through excited-state-altering response from intramolecular charge transfer (ICT) to local excitation (LE) state. Two probes, based on the tricyanofuran and naphthol units, demonstrated remarkable sensitivity and selectivity for *β*-Gal, with a low LOD value of 0.003 U mL^−1^ for fluorescence and 0.0002 U mL^−1^ for absorption. The unique optical properties of probes were dominated by ICT in their initial state and shifted to LE upon *β*-Gal response, allowing for the ratiometric absorption and activatable fluorescence changes and providing a robust platform for *β*-Gal detection and quantification. Finally, the probes were employed for the 24 h long period of fluorescence sensing targeting *β*-Gal in senescent and normal A549 cells, confirming the overexpression of *β*-Gal during cellular senescence. Overall, the development of **TF1** and **TF2** represents a significant advancement in the field of fluorescence analysis for senescent cell detection. These probes not only offer a sensitive and selective tool for *β*-Gal detection but also have the potential to contribute to the understanding of cellular senescence and its associated diseases. Future efforts will focus on optimizing biocompatibility and extending applications to in vivo aging models.

## Data Availability

Data are contained within the article and Supplementary Materials.

## Supplementary Materials

The following supporting information can be downloaded at https://www.mdpi.com/article/10.3390/molecules30061221/s1. Scheme S1: Synthesis procedures of probes TF1 and TF2; Scheme S2: Structure list of compound TF1, TF1-OH, TF1-O, TF2, TF2-OH and TF2-O; Scheme S3: Structure variation of TF1 and TF2 after incubating *β*-Gal and then adding CF_3_COOH; Scheme S4: Speculated structural transformation of local excited dyes TF1-O and TF2-O between naphthoxide anions and naphthoquinone types; Table S1: Photophysical properties of TF1 and TF2 in various solvents; Tables S2–S4: Optimized geometries coordinate of TF1, TF2, TF1-OH, TF2-OH, TF1-O and TF2-O; Figure S1: Photos of TF-O and TF-OH from *β*-Gal-cut (a) TF1 and (b) TF2 after adding with or without trifluoroacetic acid; Figure S2: Mass spectra of residues (a) TF1-OH and (b) TF2-OH; Figure S3: Calculated highest occupied molecular orbitals (HOMOs) and lowest unoccupied molecular orbitals (LUMOs) of TF1, TF2, TF1-OH, TF2-OH, TF1-O and TF2-O by using density functional theory at B3LYP/6-31G(d,p) level of theory in gas phase; Figure S4: Calculated overlapped distribution of holes (blue) and electrons (green) of TF1, TF2, TF1-OH, TF2-OH, TF1-O and TF2-O in excited state by using density functional theory at CAM-B3LYP/6-31g(d) level of theory in gas phase; Figure S5: Relative (a) absorption and (b) fluorescence spectra of TF1 incubated with *β*-Gal at as function of time; Figure S6: Relative (a) absorption and (b) fluorescence spectra of TF2 incubated with *β*-Gal at as function of time; Figure S7: Absorption ratio of (a) TF1 and (b) TF2 incubated with *β*-Gal at as function of time; Figure S8: (a) Fluorescence spectra of TF1 incubated with *β*-Gal and other analytes for 1 h; (b) Relative absorption spectra and (c) corresponding absorption intensity comparison of TF1 with *β*-Gal and other analytes for 1 h; Figure S9: (a) Fluorescence spectra of TF2 incubated with *β*-Gal and other analytes for 1 h; (b) Relative absorption spectra and (c) corresponding absorption intensity comparison of TF2 with *β*-Gal and other analytes for 1 h; Figure S10: Ratiometric absorption spectra of (a) TF1 and (b) TF2 incubated with different concentration of *β*-Gal; Plotting the (c) *A*_632_/*A*_475_ of TF1 and (d) the *A*_631_/*A*_460_ TF2 incubated with different concentration of *β*-Gal; Figure S11: Cell viability of (a) normal and (b) senescent A549 cells after incubation with different concentrations of TF1 and TF2 for 24 h; Figure S12: (a) Fluorescence imaging and (b) quantitative signal intensity of senescent A549 cells after incubating with different concentrations of TF1 for 4 h; Figure S13: (a) Fluorescence imaging and (b) quantitative signal intensity of senescent A549 cells after incubating with different concentrations of TF2 for 4 h; Figure S14: (a) Fluorescence imaging and (b) quantitative signal intensity of senescent A549 cells after incubating TF1 with different time; Figure S15: (a) Fluorescence imaging and (b) quantitative signal intensity of senescent A549 cells after incubating TF2 with different time; Figure S16: Fluorescence co-localization of TF1 with (a) LysoTracker and (b) MitoTracker; Figure S17: Fluorescence co-localization of TF2 with (a) LysoTracker and (b) MitoTracker; Figure S18: ^1^H-NMR of compound TF1-OH in DMSO-*d*_6_; Figure S19: ^1^H-NMR of compound TF1-GalAc in CDCl_3_; Figure S20: ^1^H-NMR and ^13^C-NMR of compound TF1 in DMSO-*d*_6_; Figure S21: ^1^H-NMR of compound TF2-OH in DMSO-*d*_6_; Figure S22: ^1^H-NMR of compound TF2-GalAc in CDCl_3_; Figure S23: ^1^H-NMR and ^13^C-NMR of compound TF2 in DMSO-*d*_6_; Figure S24: HRMS spectral of TF1; Figure S25: HRMS spectral of TF2.
