# Supplementary material for: Excited-State-Altering Ratiometric Fluorescent Probes for the Response of β-Galactosidase in Senescent Cells
Source: Molecules. 2025 Mar 8;30(6):1221. doi: 10.3390/molecules30061221 (PMC11945828; doi:10.3390/molecules30061221)
Supplement: Supplementary file 1 [file molecules-30-01221-s001.zip › molecules-3457272-supplementary.pdf]

Supporting Information

Excited-State-Altering Ratiometric Fluorescent Probes for the Response of *β*-Galactosidase in Senescent Cells

Ya-Nan Han [1,†], Lei Dong [3,*,†], Lu-Lu Sun [2,†], Wen-Jia Li [2,4], Jianjing Xie [1], Congyu Li [1], Shuhui Ren [1], Zhan Zhang [5], Hai-Hao Han [2,4,*] and Zhong Zhang [1,*]

[1] School of Life Science and Technology, Shandong Second Medical University, Weifang 261053, China; hanyanan0911@163.com (Y.-N.H.); xiejianjing955@163.com (J.X.); licongyua@163.com (C.L.); rensh1012@163.com (S.R.)
[2] Shandong Laboratory of Yantai Drug Discovery, Bohai Rim Advanced Research Institute for Drug Discovery, Yantai 264117, China; llsun@baridd.ac.cn (L.-L.S.); liwenjia@simm.ac.cn (W.-J.L.)
[3] School of Pharmacy, Shandong Second Medical University, Weifang 261053, China
[4] Molecular Imaging Center, Stake Key Laboratory of Chemical Biology, Shanghai Institute of Materia Medica, Chinese Academy of Sciences, Shanghai 201203, China
[5] School of Chemistry and Chemical Engineering, Yangzhou University, Yangzhou 225002, China; zhanzhang@yzu.edu.cn
***** Correspondence: leidong@sdsmu.edu.cn (L.D.); hanhaihao@simm.ac.cn (H.-H.H.); nasonia@163.com (Z.Z.)

[†] These authors contributed equally to this work.

Contents List

Synthesis of photosensitizers **TF1** and **TF2** S2-S4

Experimental section S5-S6

Additional figures and schemes S7-S24

References S25

Synthesis of photosensitizers TF1 and TF2

General methods

All reagents for synthesis were commercially available (highest purity available for reagent grade compounds) and were used without further purification. Reactions under microwave activation were performed on a Biotage Initiator system. Thin-layer chromatography (TLC) was carried out on aluminum sheets coated with silica gel 60 F_{254} (Merck). TLC plates were inspected by UV light (λ = 254 nm, 365 nm) and developed by treatment with a mixture of 10% H_2SO_4 in EtOH/H_2O (95:5 v/v) followed by heating. Silica gel column chromatography was performed with silica gel Si 60 (40–63 µm). NMR spectra were recorded at 293 K, unless stated otherwise. Chemical shifts are referenced relative to deuterated solvent residual peaks. The following abbreviations were used to explain the observed multiplicities: s, singlet; d, doublet; t, triplet; q, quadruplet; m, multiplet; p, pseudo; and b, broad.

Synthesis procedures

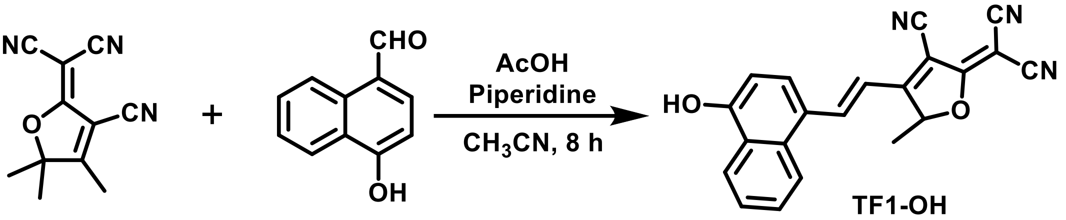

*Synthesis procedure of compound **TF1-OH***

Piperidine (0.3 mL) and AcOH (0.3 mL) were added to a solution of 4-hydroxy-1-naphthaldehyde (500 mg, 2.90 mmol, 1 eq.) and 2-(3-cyano-4,5,5-trimethylfuran-2(*5H*)-ylidene)malononitrile [1] (TCF, 695 mg, 3.48 mmol, 1.2 eq.) in CH_3CN (25 mL). The mixture was refluxed until TCL identified that the starting materials had disappeared. The reaction was diluted with EtOAc (50 mL) and washed with HCl aqueous solution (2M, 40 mL) and brine (40 mL × 3). The combined organic layer was dried (Na_2SO_4), concentrated, and purified with silica gel column chromatography (CH_2Cl_2:EtOAc = 15:1, v/v) to afford compound **TF1-OH** (674 mg, 68%) as a red powder.

^{1}H NMR (600 MHz, DMSO-*d*$_6$): δ (ppm) 8.84 (d, *J* = 15.8 Hz, 1H), 8.34 (d, *J* = 8.4 Hz, 1H), 8.27 (d, *J* = 8.2 Hz, 1H), 8.22 (d, *J* = 8.6 Hz, 1H), 7.72 (t, *J* = 7.6 Hz, 1H), 7.62 – 7.54 (m, 1H), 7.22 (d, *J* = 15.8 Hz, 1H), 7.07 (fd, *J* = 8.3 Hz, 1H), 1.80 (s, 6H).

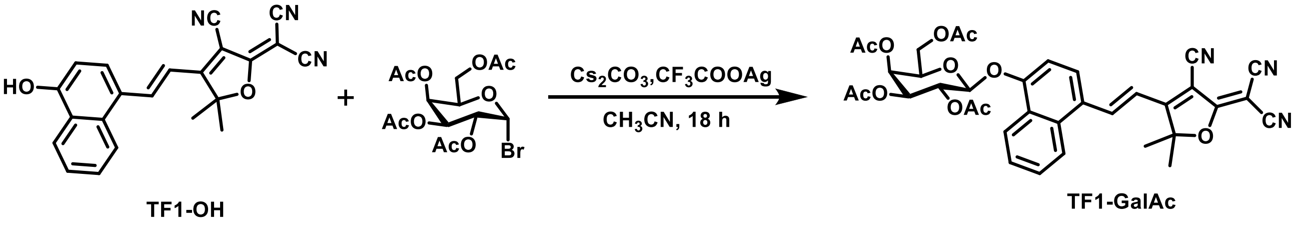

*Synthesis procedure of compound **TF1-GalAc***:

Cs_2CO_3 (738 mg, 2.26 mmol, 2 eq.) and sliver trifluoroacetate (CF_3COOAg, 500 mg, 2.26 mmol, 2 eq.) were added to a solution of **TF1-OH** (400 mg, 1.13 mmol, 1 eq.) and 2,3,4,6-tetra-*O*-acetyl-α-D-galactopyranosyl bromide (559 mg, 1.36 mmol, 1.2 eq.) in CH_3CN (30 mL). The resulting mixture was stirred at room temperature for 18 h. The reaction was diluted with EtOAc (50 mL) and washed with brine (40 mL × 3). The combined organic layer was dried (Na_2SO_4), concentrated, and purified with silica gel column chromatography (CH_2Cl_2:MeOH = 50:1, v/v) to afford compound **TF1-GalAc** (428 mg, 55%) as a yellow powder.

^{1}H NMR (600 MHz, $CDCl_3$): δ (ppm) 8.59 (d, *J* = 16.1 Hz, 1H), 8.24 (d, *J* = 8.4 Hz, 1H), 8.05 (d, *J* = 8.5 Hz, 1H), 7.97 (d, *J* = 8.3 Hz, 1H), 7.70 (t, *J* = 7.7 Hz, 0H), 7.65 – 7.61 (m, 2H), 7.15 (d, *J* = 8.3 Hz, 1H), 7.06 (d, *J* = 16.1 Hz, 1H), 5.74 (dd, *J* = 10.5, 7.9 Hz, 1H), 5.54 (d, *J* = 3.4 Hz, 1H), 5.34 (d, *J* = 8.0 Hz, 1H), 5.22 (dd, *J* = 10.6, 3.5 Hz, 1H), 4.29-4.26 (m 1H), 4.24 – 4.19 (m, 2H), 4.12 (q, *J* = 7.1 Hz, 1H), 2.22 (s, 3H), 2.11 (s, 3H), 2.06 (s, 3H), 2.04 (s, 3H), 1.87 (s, 6H).

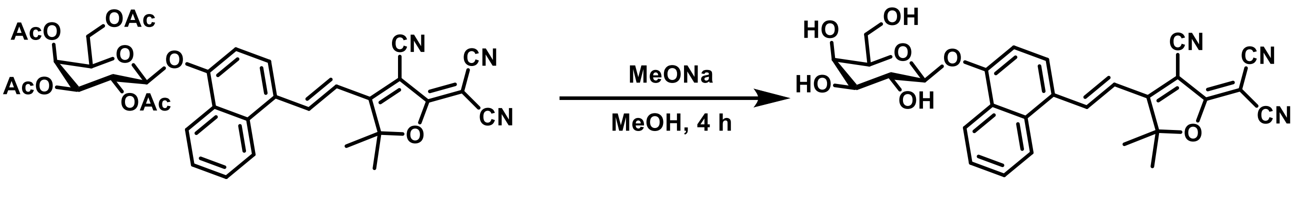

Synthesis procedure of compound ***TF1***:

The glycosylated compound **TF1-GalAc** (170 mg, 0.249 mmol, 1 eq.) was sufficiently dissolved in MeOH (30 mL) mixed with tetrahydrofuran (THF, 2-3 mL). Sodium methoxide (MeONa, 81 mg, 1.49 mmol, 6 eq.) was subsequently added to the solution, which was then stirred at room temperature for 4 hours. Thin-layer chromatography (TLC) was employed to monitor the reaction until the starting materials had completely disappeared. The reaction was neutralized with 2 M HCl aqueous solution, evaporated to dryness, and purified using column chromatography (CH_2Cl_2:MeOH = 10:1, v/v) to obtain the targeting compound **TF1** as an amorphous dark-red solid (76 mg, 60%).

^{1}H NMR (400 MHz, DMSO-d_6): *δ* (ppm) 8.85 (d, *J* = 16.0 Hz, 1H), 8.46 (d, *J* = 8.3 Hz, 1H), 8.38 (d, *J* = 8.4 Hz, 1H), 8.25 (d, *J* = 8.6 Hz, 1H), 7.76 (t, *J* = 7.7 Hz, 1H), 7.65 (t, *J* = 7.7 Hz, 1H), 7.35 (s, 1H), 7.34 (d, *J* = 25.8 Hz, 1H), 5.43 (d, *J* = 5.3 Hz, 1H), 5.17 (d, *J* = 7.7 Hz, 1H), 4.96 (d, *J* = 5.7 Hz, 1H), 4.70 (t, *J* = 5.4 Hz, 1H), 4.61 (d, *J* = 4.6 Hz, 1H), 3.85-3.72 (m, 3H), 3.59-3.51 (m, 3H), 1.82 (s, 6H).

^{13}C NMR (101 MHz, DMSO-d_6): *δ* (ppm) 25.0, 53.8, 60.5, 68.2, 70.3, 73.1, 76.0, 96.7, 99.4, 101.1, 109.1, 112.0, 112.9, 115.0, 122.3, 123.2, 124.1, 125.1, 126.2, 128.6, 128.8, 131.6, 131.7, 132.4, 143.0, 157.1, 175.8, 177.5.

HR-ESI-MS *m/z*: calcd. for $C_{28}H_{25}N_3O_7Na$ $[M+Na]^+$ 538.1590 found 538.1594.

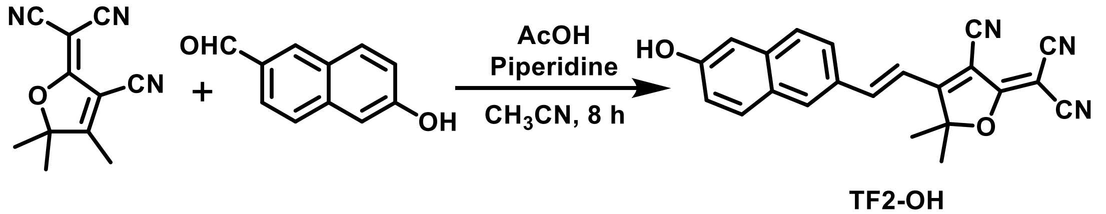

Synthesis procedure of compound ***TF2-OH***

Piperidine (0.3 mL) and AcOH (0.3 mL) were added to a solution of 6-hydroxy-2-naphthaldehyde (500 mg, 2.90 mmol, 1 eq.) and TCF (695 mg, 3.48 mmol, 1.2 eq.) in CH_3CN (25 mL). The mixture was refluxed until monitoring with TCL identified that the starting materials had completely disappeared. The reaction was diluted with EtOAc (50 mL), washed with HCl aqueous solution (2M, 40 mL) and brine (40 mL × 3). The combined organic layer was dried (Na_2SO_4), concentrated, and purified with silica gel column chromatography (CH_2Cl_2:EtOAc = 15:1, v/v) to afford compound **TF2-OH** (505 mg, 51%) as a red powder.

^{1}H NMR (600 MHz, DMSO-d_6): δ (ppm) 10.32 (s, 1H), 8.31 (s, 1H), 8.09 (d, *J* = 16.3 Hz, 1H), 7.96 (d, *J* = 7.4 Hz, 1H), 7.91 (d, *J* = 8.8 Hz, 1H), 7.79 (d, *J* = 8.8 Hz, 1H), 7.25 (d, *J* = 16.3 Hz, 1H), 7.19 (d, *J* = 2.1 Hz, 2H), 7.17 (dd, *J* = 8.8, 2.3 Hz, 1H), 1.82 (s, 6H).

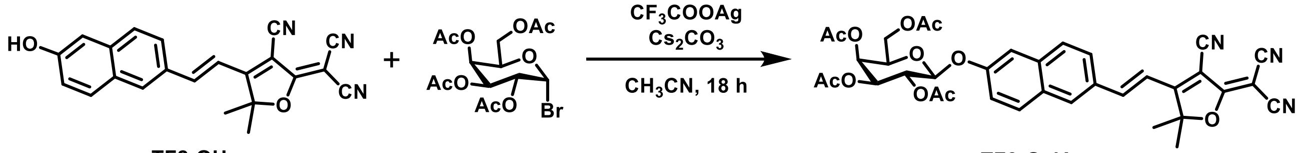

TF2-OH TF2-GalAc

*Synthesis procedure of compound **TF2-GalAc***:

Cs_2CO_3 (645 mg, 1.98 mmol, 2 eq.) and sliver trifluoroacetate (CF_3COOAg, 438 mg, 1.98 mmol, 2 eq.) were added to a solution of **TF2-OH** (350 mg, 0.990 mmol, 1 eq.) and 2,3,4,6-tetra-*O*-acetyl-α-D-galactopyranosyl bromide (489 mg, 1.19 mmol, 1.2 eq.) in CH_3CN (30 mL). The resulting mixture was stirred at room temperature for 18 h. The reaction was diluted with EtOAc (50 mL) and washed with brine (40 mL × 3). The combined organic layer was dried (Na_2SO_4), concentrated, and purified with silica gel column chromatography (CH_2Cl_2:MeOH = 50:1, v/v) to afford compound **TF2-GalAc** (452 mg, 67%) as a yellow powder.

^{1}H NMR (400 MHz, $CDCl_3$): δ (ppm) 8.02 (s, 1H), 7.86 (d, *J* = 9.0 Hz, 1H), 7.83 – 7.72 (m, 1H), 7.37 (d, *J* = 2.2 Hz, 1H), 7.29 (d, *J* = 2.4 Hz, 1H), 7.12 (d, *J* = 16.4 Hz, 1H), 5.57 (dd, *J* = 10.4, 7.9 Hz, 1H), 5.51 (d, *J* = 3.3 Hz, 1H), 5.26 (d, *J* = 8.0 Hz, 1H), 5.17 (dd, *J* = 10.4, 3.5 Hz, 1H), 4.30 – 4.25 (m, 1H), 4.22 – 4.15 (m, 2H), 2.20 (s, 3H), 2.08 (s, 3H), 2.08 (s, 3H), 2.03 (s, 3H), 1.83 (s, 6H).

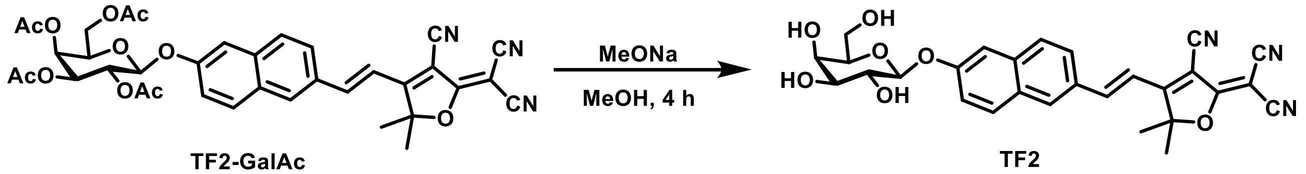

TF2-GalAc TF2

*Synthesis procedure of compound **TF2***:

The glycosylated compound **TF2-GalAc** (160 mg, 0.234 mmol, 1 eq.) was sufficiently dissolved in MeOH (30 mL) mixed with tetrahydrofuran (THF, 2-3 mL). Sodium methoxide (MeONa, 76 mg, 1.40 mmol, 6 eq.) was subsequently added to the solution, which was then stirred at room temperature for 4 hours. Thin-layer chromatography (TLC) was employed to monitor the reaction until the starting materials had completely disappeared. The reaction was neutralized with 2 M HCl aqueous solution, evaporated to dryness, and purified using column chromatography (CH_2Cl_2:MeOH = 10:1, v/v) to obtain the targeting compound **TF2** as an amorphous dark-red solid (54 mg, 65%).

^{1}H NMR (600 MHz, DMSO-d_6): δ (ppm) 8.86 (d, *J* = 16.0 Hz, 1H), 8.46 (d, *J* = 8.5 Hz, 1H), 8.39 (d, *J* = 8.5 Hz, 1H), 8.25 (d, *J* = 8.8 Hz, 1H), 7.76 (t, *J* = 7.0 Hz, 1zH), 7.67-7.64 (m, 1H), 7.37 – 7.31 (m, 2H), 5.18 (d, *J* = 7.7 Hz, 2H), 3.82 (t, *J* = 8.6 Hz, 1H), 3.77 (d, *J* = 3.0 Hz, 1H), 3.74 (t, *J* = 6.3 Hz, 1H), 3.61 – 3.49 (m, 3H), 1.82 (s, 6H).

^{13}C NMR (150 MHz, DMSO-d_6): δ (ppm) 25.4, 54.2, 60.9, 68.7, 70.8, 73.6, 76.4, 97.1, 99.9, 101.5, 109.5, 112.5, 113.3, 115.4, 122.8, 123.7, 124.6, 125.6, 126.6, 129.1, 129.2, 132.8, 143.5, 157.6, 176.3, 178.0.

HR-ESI-MS *m/z*: calcd. for $C_{28}H_{25}N_3O_7Na$ $[M+Na]^+$ 538.1590 found 538.1595.

Experimental section

Material and instruments. Materials were obtained from commercial suppliers and were used without further purification. All reactions were performed in oven-dried glassware (Hinwil) unless otherwise stated. Column chromatography was performed over silica gel (200-300 mesh). NMR spectra were recorded with JEOL-400 or JEOL-600 spectrometers. High-resolution mass spectrometry experiments were recorded by Bruker Solarix XR Fourier Transform Ion Cyclotron Resonance Mass Spectrometer. All optical spectra were recorded at room temperature.

UV-Vis and fluorescence spectroscopy. UV-visible spectra were analyzed using a Hitachi UV-2550 spectrophotometer. All spectra were corrected for background intensities by subtracting the spectra of pure solvent measured under identical conditions. The fluorescence measurements were carried out at room temperature using Hitachi F-4600 spectrophotometer. The excitation slit width was 5 nm, emission slit width was 10 nm, and PMT voltage was 700V. The fluorescence of residue was excited at 570 nm, and the emission signal was collected at 590–750 nm. The relative absorption (*Abs*) and relative emission intensity (I_F) were normalized by setting the maximum values of the spectra to 1, with all other values scaled proportionally within the [0,1] range.

Absolute fluorescence quantum yield. TF1-O, **TF1-OH**, **TF2-O** and **TF2-OH** were measured in PBS buffer mixed with 10% DMSO with diluted concentration as 10 µM. The absolute fluorescence quantum yield was determined by Hamamatsu Quantaurus-QY.

Calculation of optimized geometries and HOMO-LUMO. Gaussian 16 program was used to perform density functional theory (DFT) calculations (B3LYP/6-31G(d,p)) of **TF1**, **TF2**, and their derivatives in the gas phase. The high-performance computing server was provided by Beijing Super Cloud Computing Center, Beijing, China.

Calculation of natural transmission orbitals (NTOs). Gaussian 16 program calculated the NTOs by using density functional theory at PBE1PBE/6-31G* level of theory in the gas phase. The software program Multiwfn was used to analyze the NTO results and generate graphs of hole-particle distribution [2-5]. The high-performance computing server was provided by Beijing Super Cloud Computing Center, Beijing, China.

Calculation of hole-electron distribution in excited state. Gaussian 16 program calculated the hole-electron distributions in S1 excited state by using density functional theory at CAM-B3LYP/6-31G(d) level of theory in the gas phase. The software program Multiwfn was used to analyze the calculation results and generate graphs of hole–electron distribution [2-5]. The high-performance computing server was provided by Beijing Super Cloud Computing Center, Beijing, China.

***β*-Gal hydrolysis in solution.** 10 µL DMSO solution of probe (**TF1** or **TF2**, 2 mM**)** and 10 µL PBS solution of *β*-Gal (800 U mL^{-1}) were added into 1980 µL PBS buffer (pH 7.4, 1 mM) mixed with 10% DMSO to prepare the testing sample (2 mL) with concentrations of probe and *β*-Gal as 10 µM and 4 U mL^{-1}, respectively. The resulting sample was placed at 37 °C for 1 h before measured the absorption and fluorescence spectra.

Cell viability

The normal and senescent A549 cells were seeded on 96-well plates (5×10^3 cells $well^{-1}$) and incubated overnight. Then, the medium was replaced with 100 µL of 1640 (contain 1% DMSO) containing different concentrations (0-40 µM) of **TF1** or **TF2**. After incubation for another 24 h, the cell viability was examined using a cell counting kit-8

(CCK-8) assay.

Cellular evaluation of TF1 and TF2 responses at various concentrations and time points in senescent A549 Cells

To study the response of the **TF1** and **TF2** probes, senescent A549 cells were seeded onto 24-well plates and incubated with the **TF1** or **TF2** probe at indicated concentrations (0, 1, 2, 5, 10, 20 μM) for 4 h or incubated with 10 μM **TF1** or **TF2** probes for indicated time points (0, 2, 4, 6, 18, 24 h). Cells were fixed with 4% PFA and washed with phosphate buffered saline (PBS) three times before imaging. A laser scanning confocal microscopy (Olympus-FV3000, Olympus, Tokyo, Japan) was used to image at E_x = 640 nm and E_m = 650-700 nm.

Additional figures and schemes

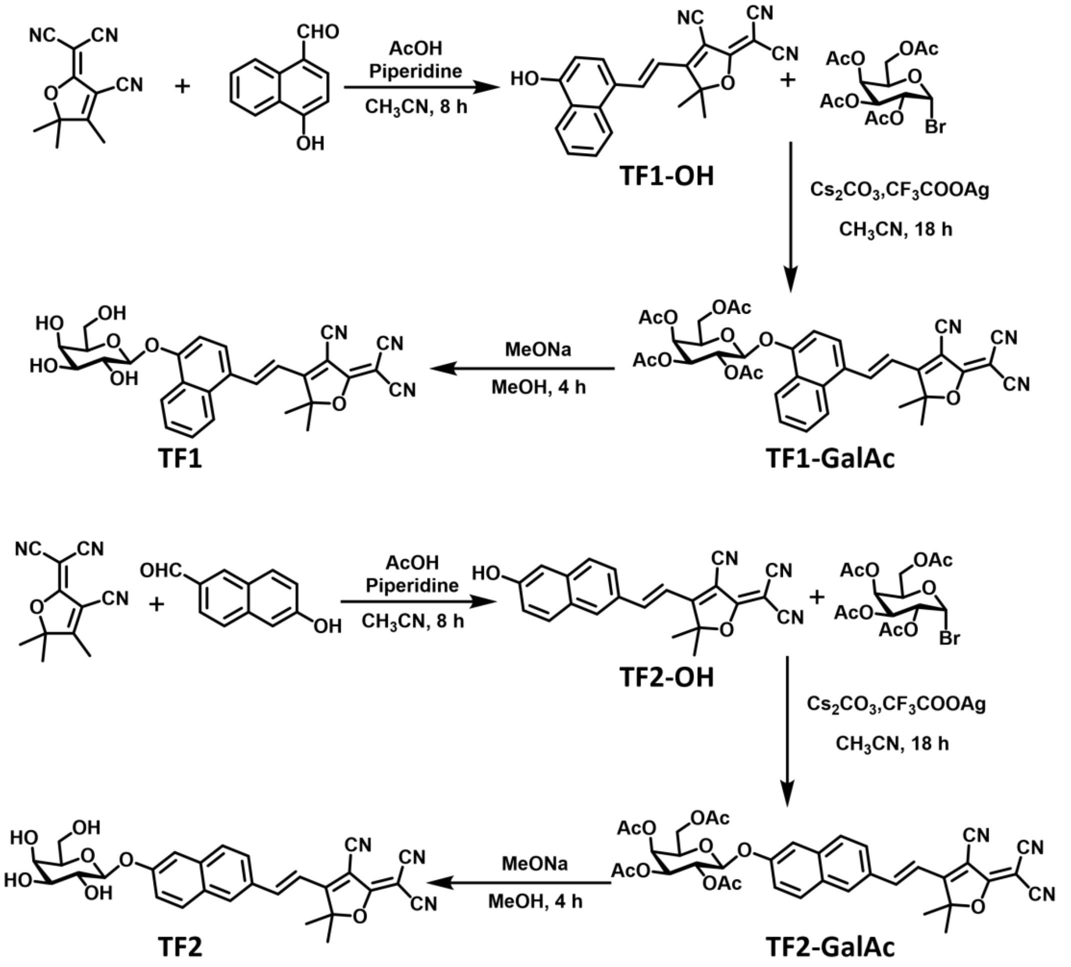

Scheme S1. Synthesis procedures of probes **TF1** and **TF2**.

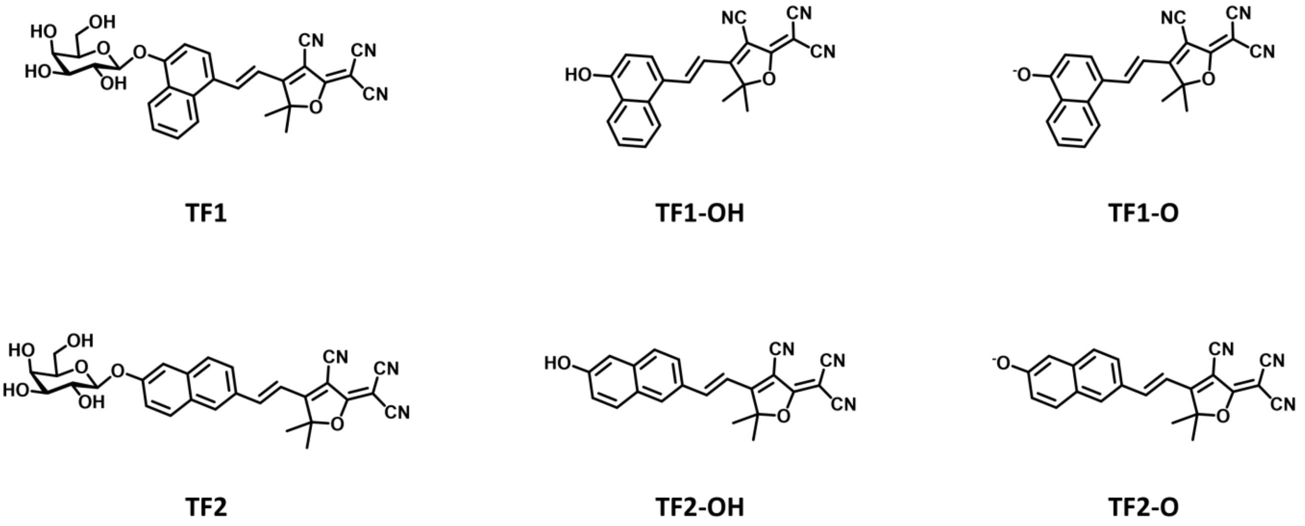

Scheme S2. Structure list of compounds **TF1**, **TF1-OH**, **TF1-O**, **TF2**, **TF2-OH** and **TF2-O**.

Table S1. Photophysical properties of **TF1** and **TF2** in various solvents

Name	λ_{abs} (nm)[1]	ε (mol^{-1} cm^{-1})	λ_{em} (nm)[2]	Stokes-shift (nm)	Φ (%)[3]	HOMO (eV)	LUMO (eV)	ΔE(L-H) (eV)[4]	ΔE(S_1-S_0) (eV)
**TF1**	475	11400	613	138	--	-5.291	-3.178	2.74	2.79
**TF1-OH**	487	20200	633	146	1.59	-5.968	-3.227	2.74	2.84
**TF1-O**	632	80100	661	30	1.25	-2.330	-0.154	2.18	2.62
**TF2**	460	11900	604	144	--	-6.064	-3.259	2.81	2.73
**TF2-OH**	487	20000	634	147	1.34	-6.066	-3.277	2.79	2.75
**TF2-O**	631	52000	659	29	1.03	-2.292	-0.175	2.12	2.26

[1]Wavelength of absorption maxima. [2]Wavelength of fluorescence maxima. [3]Absolute quantum yields of fluorescence were measured in PBS buffer mixed with 10% DMSO. [4]ΔE(L-H) is the energy gap between HOMO and LUMO levels.

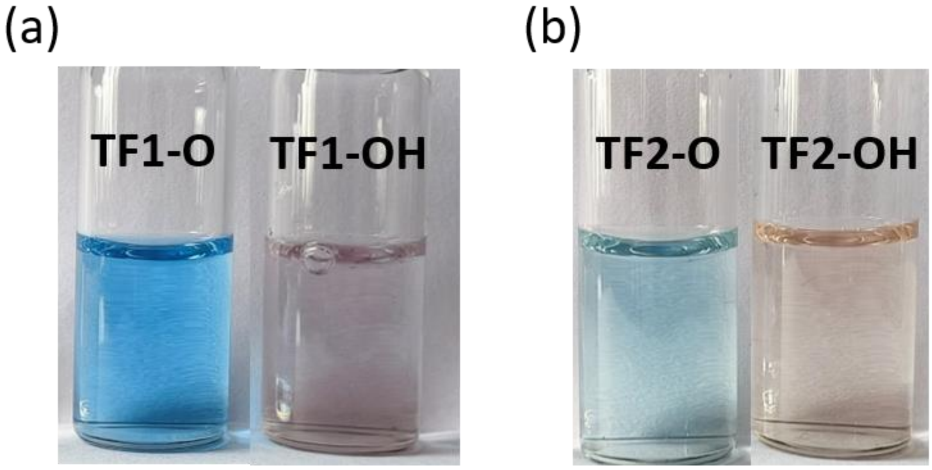

Figure S1. Photos of **TF-O** (left) and **TF-OH** (right) from *β*-Gal-cut (a) **TF1** and (b) **TF2** (10 μM) after adding with (right) or without (left) trifluoroacetic acid (50 μL).

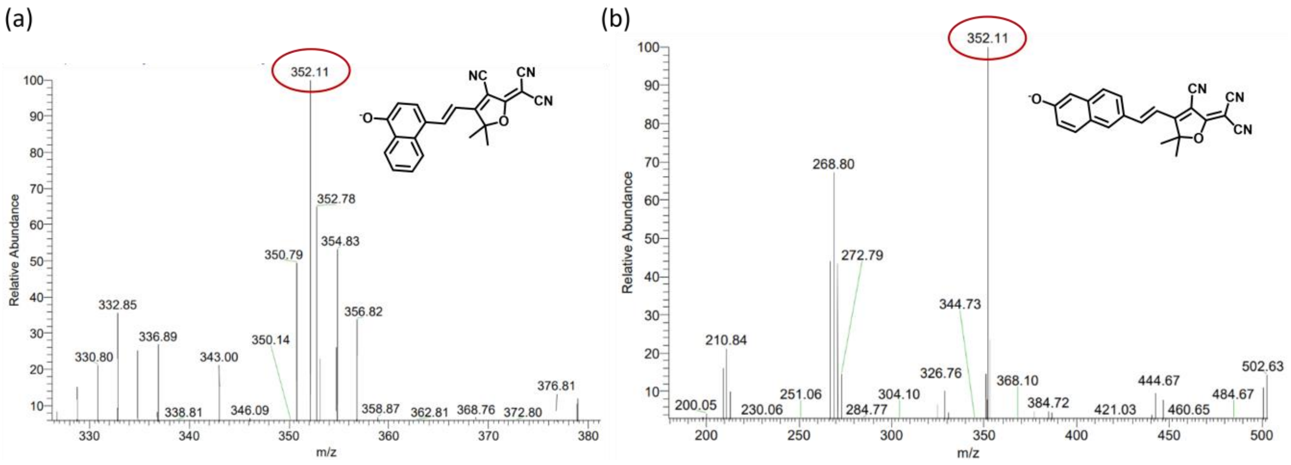

Figure S2. Mass spectra of residues (a) **TF1-OH** and (b) **TF2-OH**.

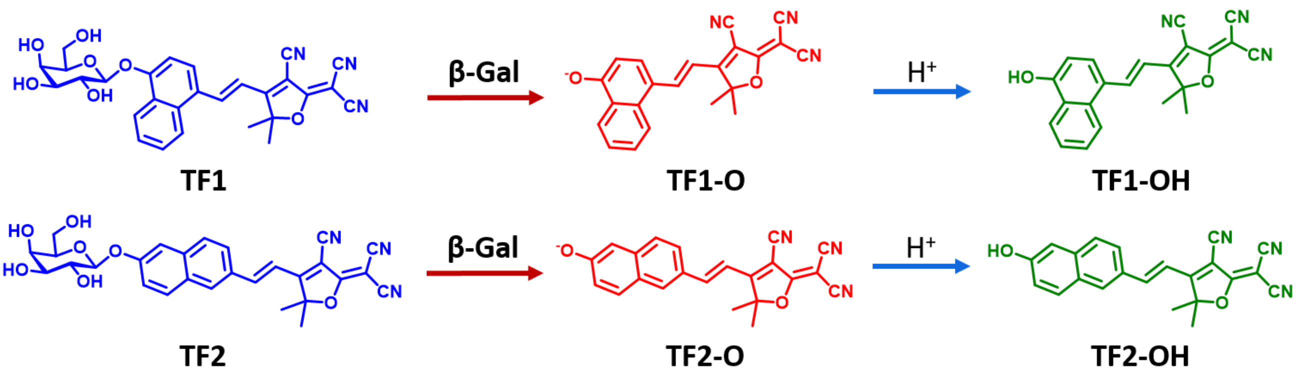

Scheme S3. Structure variation of **TF1** and **TF2** after incubating *β*-Gal, and then adding CF_3COOH (50 µL).

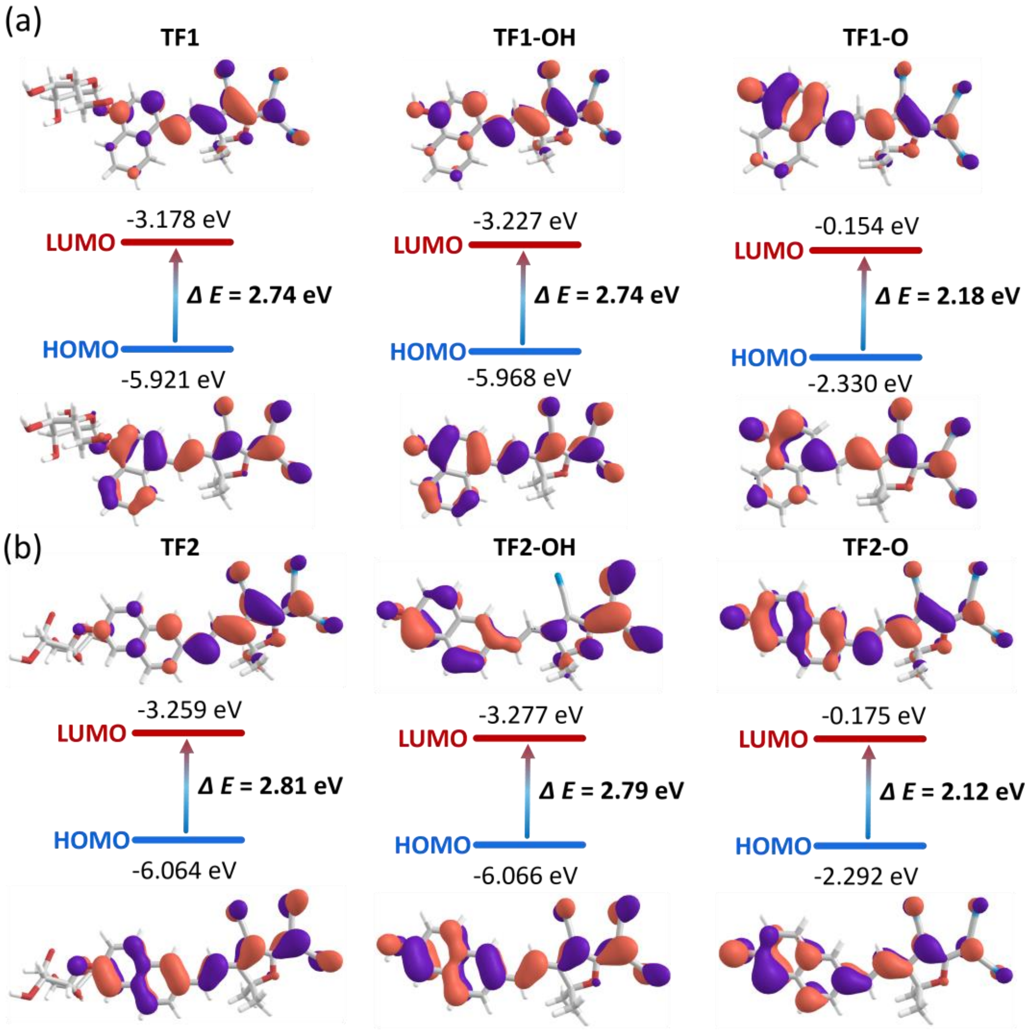

Figure S3. Calculated highest occupied molecular orbitals (HOMOs) and lowest unoccupied molecular orbitals (LUMOs) of **TF1, TF2**, **TF1-OH**, **TF2-OH**, **TF1-O,** and **TF2-O** using density functional theory at the B3LYP/6-31G(d,p) level of theory in the gas phase.

Table S2. Optimized geometries coordinate of **TF1** and **TF2**

**TF1**				**TF2**			
Element	X	Y	Z	Element	X	Y	Z
C	-5.62407	-2.85533	5.47813	C	-5.97493	2.90589	1.91092
O	-4.44121	-2.60803	5.72782	C	-6.74338	2.68402	0.62767
C	-3.82348	-1.80238	4.72419	C	-5.98826	3.27627	-0.54297
C	-4.99919	-1.63607	3.78594	C	-4.51844	2.91092	-0.55741
C	-6.03743	-2.30343	4.32137	O	-3.87794	3.06046	0.69365
C	-5.0494	-0.95771	2.62181	C	-4.50812	2.51036	1.8189
C	-3.37351	-0.50625	5.41274	C	-4.18718	1.03063	2.00166
C	-2.6636	-2.62615	4.14758	O	-2.78461	0.82976	1.98153
C	-6.32363	-3.60976	6.35046	O	-6.62451	2.17898	2.92549
C	-4.03544	-0.27813	2.05224	O	-6.93572	1.30292	0.4509
C	-4.00451	0.42391	0.89307	O	-6.10875	4.6799	-0.48576
C	-5.1135	0.5046	0.13461	O	-4.33373	1.60005	-1.03706
C	-5.14836	1.16844	-1.0251	O	6.74674	-2.75394	-0.27986
C	-4.07661	1.81865	-1.50618	C	5.84683	-1.65181	-0.39488
C	-2.95433	1.74895	-0.75209	C	4.53703	-2.40746	-0.41819
C	-2.90437	1.06963	0.42194	C	4.82593	-3.7177	-0.323
C	-1.85209	2.37883	-1.21179	C	6.16465	-3.84157	-0.24461
C	-0.68882	2.35838	-0.55047	C	6.18663	-0.93573	-1.70909
C	-0.62401	1.69211	0.60521	C	6.05005	-0.77945	0.85128
C	-1.7163	1.06785	1.06849	C	6.90291	-4.96543	-0.13796
O	-4.05606	2.42876	-2.73974	C	3.28966	-1.90793	-0.51542
C	-3.6173	0.71232	-5.89423	C	1.61066	1.2734	-0.80723
C	-3.52274	2.21922	-5.80877	C	1.7238	-0.06704	-0.71519
C	-4.69825	2.77275	-5.03256	C	0.57481	-0.77135	-0.73052
C	-4.96685	2.03395	-3.73778	C	-0.62952	-0.17881	-0.8349
O	-4.95229	0.62619	-3.86563	C	-0.71754	1.15909	-0.92936
C	-3.87874	0.04166	-4.55303	C	0.41742	1.87767	-0.91134
C	-2.66165	-0.18542	-3.6621	C	-1.76623	-0.89465	-0.84137
O	-3.03276	-0.86915	-2.47825	C	-2.95488	-0.28306	-0.93291
O	-2.41707	0.24417	-6.45992	C	-3.07392	1.05351	-1.03985
O	-2.30893	2.56178	-5.18815	C	-1.92087	1.75185	-1.0256
O	-5.84578	2.69567	-5.84819	C	2.95807	-0.60807	-0.6142
C	-5.73337	-4.06357	7.44361	C	6.40886	-6.18757	-0.09115
N	-5.23887	-4.47298	8.40957	N	5.9898	-7.2679	-0.04953
C	-7.5906	-3.94686	6.20535	C	8.22115	-4.87897	-0.074
N	-8.70379	-4.25094	6.09218	N	9.37852	-4.82972	-0.01677
C	-7.22876	-2.37108	3.76808	C	3.91724	-4.66849	-0.31413
N	-8.2752	-2.42331	3.2733	N	3.10942	-5.49915	-0.30811
H	-6.02355	-0.98098	2.11427	H	-6.04987	3.98335	2.19393
H	-2.87563	0.20323	4.71813	H	-7.76412	3.1289	0.70131
H	-4.23858	0.02353	5.87294	H	-6.46869	2.97295	-1.50302

H	-2.64468	-0.72753	6.22538	H	-4.01211	3.59752	-1.27792
H	-2.10777	-2.09157	3.34818	H	-4.00609	3.00345	2.69373
H	-1.92545	-2.8735	4.94414	H	-4.63939	0.37493	1.2327
H	-3.02947	-3.58701	3.719	H	-4.5402	0.6686	2.99393
H	-3.12216	-0.32808	2.64868	H	-2.44137	0.92266	1.08696
H	-6.06239	0.01878	0.40636	H	-6.23833	2.37572	3.78084
H	-6.10052	1.15146	-1.57873	H	-7.24209	0.93996	1.28768
H	-1.84738	2.95121	-2.15476	H	-6.98896	4.94158	-0.76534
H	0.19844	2.88061	-0.94564	H	5.53369	-0.0584	-1.90323
H	0.32506	1.66229	1.16737	H	7.23567	-0.56191	-1.69156
H	-1.54018	0.55442	2.02433	H	6.09084	-1.62628	-2.57788
H	-4.43209	0.44181	-6.60798	H	5.3845	0.10968	0.86774
H	-3.48188	2.66683	-6.83018	H	5.85964	-1.36058	1.78233
H	-4.54681	3.85929	-4.82984	H	7.097	-0.40245	0.89907
H	-5.98545	2.33387	-3.39333	H	2.47653	-2.64875	-0.51319
H	-4.23056	-0.99673	-4.7947	H	2.50408	1.92261	-0.79932
H	-2.14281	0.74682	-3.36993	H	0.58759	-1.86923	-0.65563
H	-1.91272	-0.83174	-4.17375	H	0.38899	2.9789	-0.98132
H	-3.47888	-0.27229	-1.86932	H	-1.7459	-1.99429	-0.75204
H	-2.47649	-0.69453	-6.64631	H	-3.87039	-0.89882	-0.9001
H	-1.61296	2.03619	-5.59442	H	-1.95041	2.8513	-1.08013
H	-5.81342	3.3806	-6.51989	H	3.79674	0.10399	-0.61402

Table S3. Optimized geometries coordinate of **TF1-OH** and **TF2-OH**

**TF1-OH**				**TF2-OH**			
Element	X	Y	Z	Element	X	Y	Z
C	-1.0105	-2.08021	3.39705	O	-7.10665	2.65266	-0.00295
O	8.10E-04	-2.57058	2.88763	O	4.06916	-1.42193	0.02264
C	0.40645	-1.88741	1.70161	C	3.13165	-0.3454	0.01641
C	-0.67278	-0.82797	1.64819	C	1.84582	-1.14133	0.01598
C	-1.47904	-1.02261	2.70749	C	2.17863	-2.44459	0.02089
C	-0.83976	0.149	0.73415	C	3.52273	-2.52847	0.02476
C	1.81698	-1.34216	1.96513	C	3.37992	0.45351	-1.27014
C	0.34732	-2.91102	0.55927	C	3.37363	0.46293	1.29829
C	-1.49872	-2.63504	4.52545	C	4.2988	-3.6317	0.03057
C	-0.05852	0.3779	-0.33898	C	0.58041	-0.67903	0.0119
C	-0.15842	1.33246	-1.29635	C	-1.2095	2.45467	-5.60E-04
C	-1.16176	2.23156	-1.23776	C	-1.05056	1.1156	0.00411
C	-1.31548	3.19809	-2.14933	C	-2.17613	0.37386	0.00591
C	-0.46764	3.31932	-3.17772	C	-3.40359	0.92771	0.00357
C	0.5451	2.43278	-3.26098	C	-3.53472	2.26698	-8.60E-04
C	0.70834	1.44911	-2.34022	C	-2.42468	3.02322	-0.003
C	1.39805	2.55622	-4.29813	C	-4.5154	0.17006	0.00576

C	2.43129	1.72092	-4.46099	C	-5.73103	0.7372	0.00362
C	2.61062	0.74465	-3.56605	C	-5.87626	2.07231	-7.90E-04
C	1.76164	0.62295	-2.5346	C	-4.76031	2.82089	-0.00298
O	-0.66499	4.32018	-4.08133	C	0.20376	0.61225	0.00706
C	-0.88989	-3.682	5.05695	C	3.84407	-4.86988	0.03334
N	-0.37424	-4.6004	5.54266	N	3.45903	-5.96358	0.03582
C	-2.56873	-2.21013	5.16917	C	5.61543	-3.50612	0.03402
N	-3.50741	-1.84755	5.74525	N	6.77226	-3.42246	0.03719
C	-2.52947	-0.27737	2.97437	C	1.30018	-3.42344	0.02188
N	-3.45237	0.38626	3.1991	N	0.51771	-4.27804	0.02266
H	-1.70443	0.80396	0.9088	H	-7.00143	3.61787	-0.00601
H	2.23421	-0.78421	1.10004	H	2.69159	1.31838	-1.37954
H	1.82369	-0.65689	2.84328	H	4.41685	0.85979	-1.28503
H	2.52378	-2.17468	2.18383	H	3.25867	-0.19029	-2.17104
H	0.64397	-2.48298	-0.42182	H	2.68507	1.32881	1.3979
H	1.03575	-3.76293	0.76134	H	3.24761	-0.17423	2.20323
H	-0.67951	-3.32834	0.44859	H	4.41058	0.86906	1.31543
H	0.76551	-0.33548	-0.40846	H	-0.2082	-1.44581	0.01299
H	-1.92181	2.23818	-0.44274	H	-0.33745	3.13234	-0.00233
H	-2.15353	3.91029	-2.05653	H	-2.12401	-0.72544	0.00972
H	1.2841	3.3481	-5.05767	H	-2.49422	4.12476	-0.00667
H	3.12135	1.83269	-5.31382	H	-4.45169	-0.93166	0.00946
H	3.45664	0.04585	-3.68354	H	-6.6302	0.09819	0.00556
H	2.01119	-0.21505	-1.86818	H	-4.87509	3.91831	-0.00656
H	-1.44824	4.83299	-3.82482	H	1.01883	1.35121	0.00569

Table S4. Optimized geometries coordinate of **TF1-O** and **TF2-O**

**TF1-O**				**TF2-O**			
Element	X	Y	Z	Element	X	Y	Z
C	-1.37584	-1.62635	3.24659	O	-7.25638	2.82121	-0.00551
O	-0.36186	-2.19727	2.83647	O	3.88185	-1.34212	0.02316
C	0.15531	-1.60923	1.64321	C	2.94593	-0.26471	0.01683
C	-0.86083	-0.50267	1.45646	C	1.65921	-1.06017	0.01621
C	-1.74289	-0.59086	2.46788	C	1.99027	-2.36359	0.02118
C	-0.91541	0.42251	0.47772	C	3.33435	-2.44798	0.0252
C	1.57319	-1.12066	1.97133	C	3.19763	0.53309	-1.26993
C	0.11492	-2.69838	0.56209	C	3.19089	0.5422	1.2992
C	-1.96254	-2.08812	4.36988	C	4.11134	-3.55045	0.03107
C	-0.05108	0.53899	-0.5486	C	0.39516	-0.59461	0.01188
C	-0.0292	1.43063	-1.56888	C	-1.36127	2.55784	-0.00139
C	-0.98229	2.3819	-1.6404	C	-1.2191	1.21691	0.00359
C	-1.01711	3.29135	-2.62069	C	-2.35264	0.48769	0.00527
C	-0.09285	3.29623	-3.58809	C	-3.57324	1.05651	0.00251
C	0.87204	2.35606	-3.5416	C	-3.68784	2.39739	-0.00232

C	0.9146	1.43	-2.55061	C	-2.56933	3.14087	-0.00433
C	1.80069	2.36569	-4.51934	C	-4.69446	0.3128	0.00462
C	2.79438	1.46936	-4.55232	C	-5.90311	0.89509	0.00198
C	2.85552	0.54791	-3.58615	C	-6.03084	2.23178	-0.00286
C	1.93103	0.53966	-2.6141	C	-4.90634	2.96695	-0.00497
O	-0.16345	4.23865	-4.56803	C	0.02918	0.69971	0.00685
C	-1.44261	-3.13004	4.99758	C	3.66705	-4.79273	0.0338
N	-1.01117	-4.04142	5.57079	N	3.30677	-5.89499	0.0363
C	-3.04711	-1.58276	4.92603	C	5.42783	-3.42178	0.03456
N	-3.99811	-1.16412	5.44099	N	6.58481	-3.33998	0.03779
C	-2.77124	0.21252	2.63286	C	1.11974	-3.34944	0.0221
N	-3.68351	0.90734	2.79924	N	0.36492	-4.22857	0.02289
H	-1.75281	1.13011	0.55315	H	2.51319	1.40077	-1.38087
H	2.07803	-0.64016	1.10652	H	4.23609	0.93551	-1.2841
H	1.55889	-0.38285	2.80573	H	3.07488	-0.11065	-2.17068
H	2.21695	-1.97261	2.28802	H	2.5059	1.41062	1.40043
H	0.49885	-2.35005	-0.4202	H	3.06344	-0.09517	2.20382
H	0.73997	-3.57019	0.86175	H	4.22926	0.94479	1.31587
H	-0.92402	-3.06847	0.40586	H	-0.39723	-1.35775	0.01295
H	0.73405	-0.21906	-0.51466	H	-0.48142	3.22538	-0.00306
H	-1.79409	2.48022	-0.90478	H	-2.31185	-0.61216	0.00936
H	-1.81787	4.0509	-2.63606	H	-2.62576	4.24316	-0.00827
H	1.78291	3.11035	-5.33331	H	-4.64422	-0.78961	0.00863
H	3.54756	1.48785	-5.35751	H	-6.80932	0.2659	0.00384
H	3.66732	-0.19961	-3.59637	H	-5.0073	4.06585	-0.00889
H	2.08635	-0.26308	-1.87913	H	0.85059	1.43141	0.0055

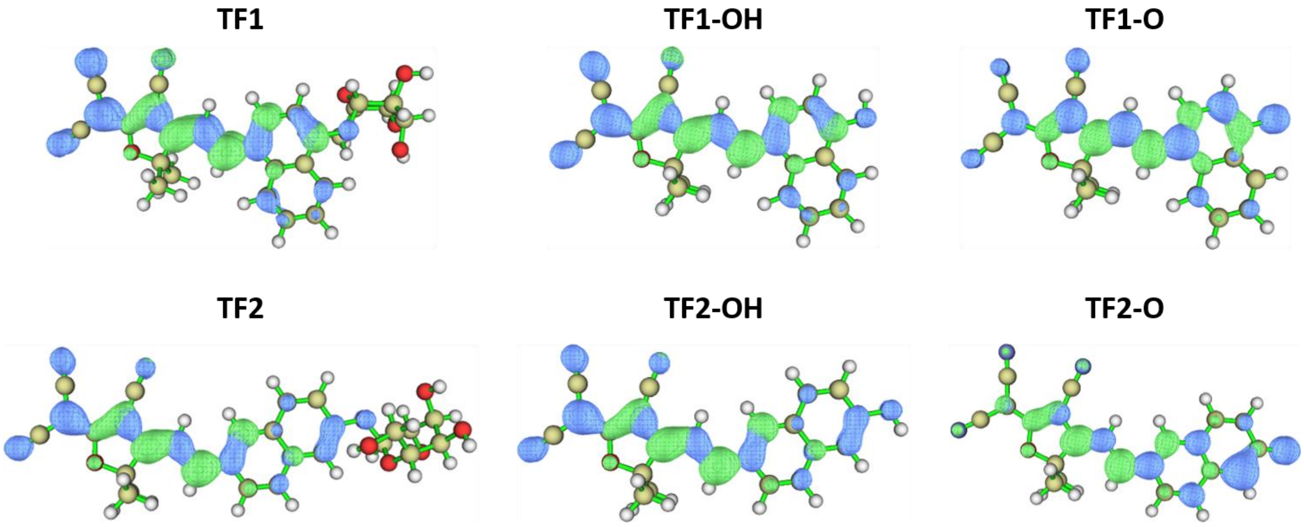

Figure S4. Calculated overlapped distribution of holes (blue) and electrons (green) of **TF1, TF2**, **TF1-OH**, **TF2-OH**, **TF1-O,** and **TF2-O** in excited state using density functional theory at the CAM-B3LYP/6-31g(d) level of theory in the gas phase.

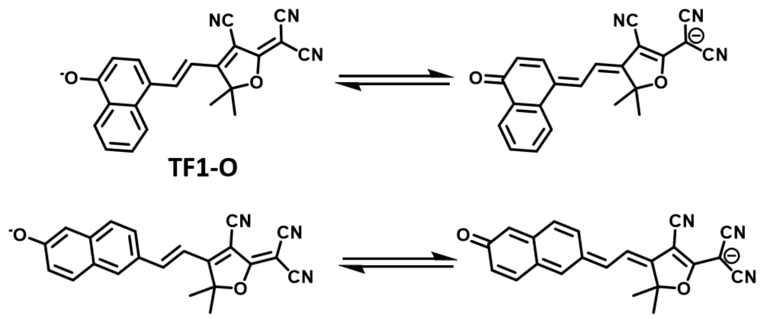

Scheme S4. Speculated structural transformation of local excited dyes TF1-O and TF2-O between naphthoxide anions (left) and naphthoquinone (right) types.

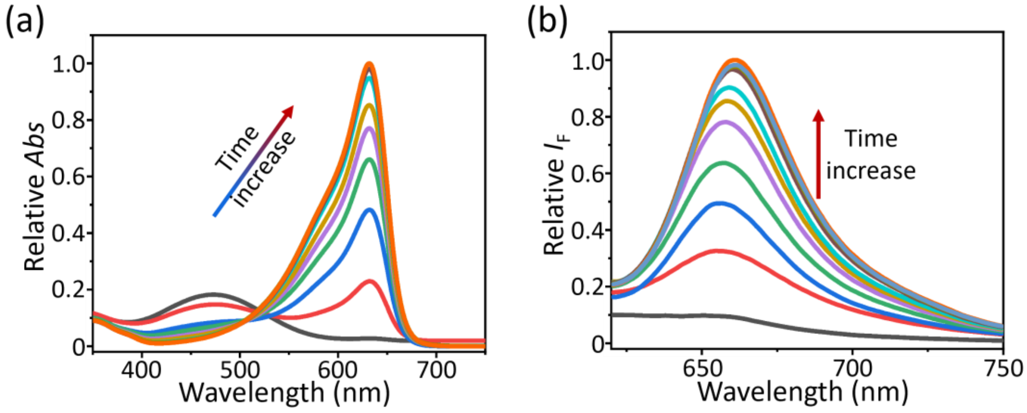

Figure S5. Relative (a) absorption and (b) fluorescence spectra of **TF1** incubated with *β*-Gal (4 U mL^{-1}) as a function of time 0–25 min (λ_{ex} = 570 nm, T = 37°C, 10% DMSO-mixed PBS, pH 7.4).

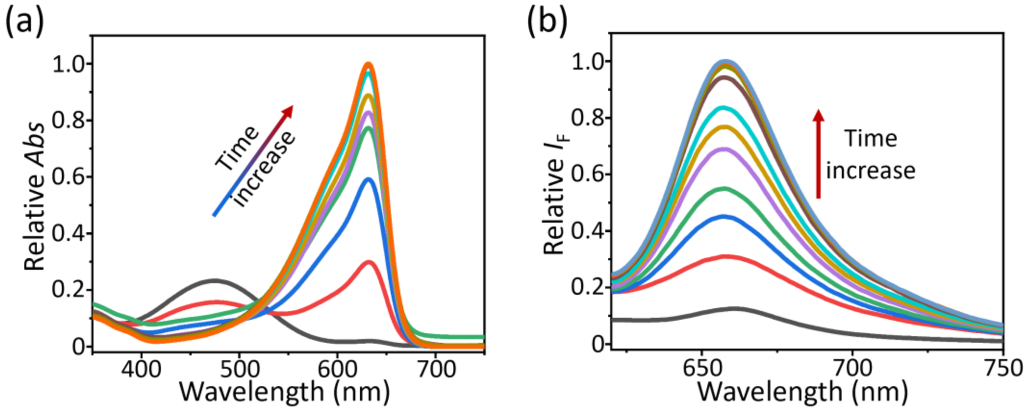

Figure S6. Relative (a) absorption and (b) fluorescence spectra of **TF2** incubated with *β*-Gal (4 U mL^{-1}) as a function of time 0–25 min (λ_{ex} = 570 nm, T = 37°C, 10% DMSO-mixed PBS, pH 7.4).

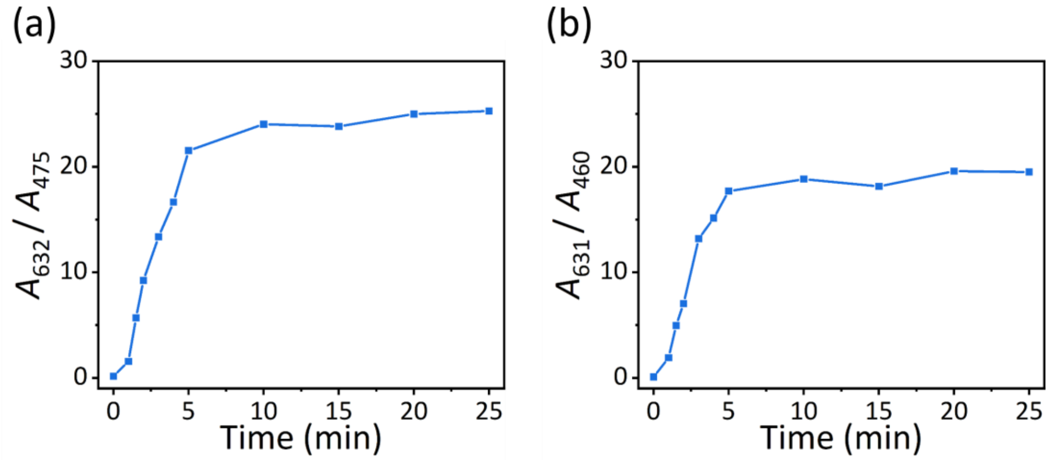

Figure S7. Absorption ratio of (a) **TF1** and (b) **TF2** incubated with *β*-Gal (4 U mL^{-1}) as a function of time 0–25 min (T = 37°C, 10% DMSO-mixed PBS, pH 7.4).

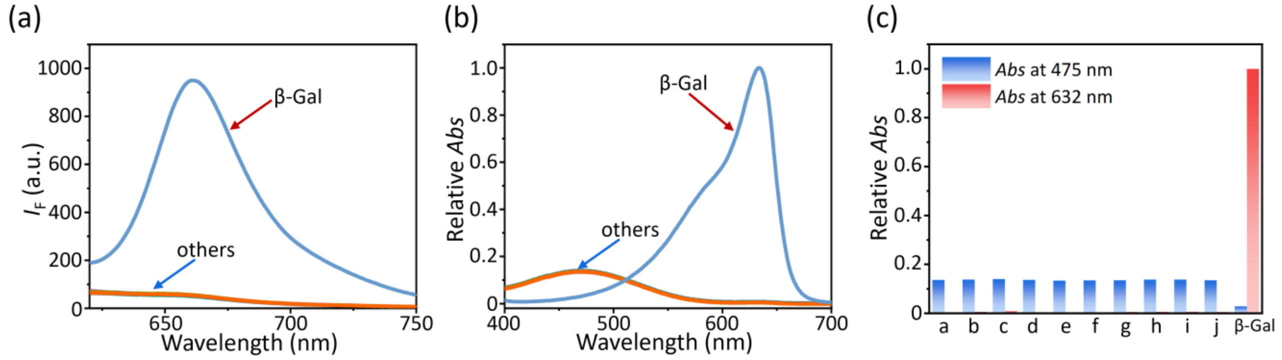

Figure S8. (a) Fluorescence spectra of **TF1** incubated with *β*-Gal (4 U mL^{-1}) and other analytes for 1 h (λ_{ex} = 570 nm, T = 37°C, 10% DMSO-mixed PBS, pH 7.4). (b) Relative absorption spectra and (c) corresponding absorption intensity comparison at 475 nm and 632 nm of **TF1** with *β*-Gal (4 U mL^{-1}) and other analytes for 1 h [a: blank, b: H_2O_2, c: NaClO, d: Hcy, e: Cys, f: GSH, (a-f, 200 μM) g: DNA, h: BSA, i: cellose (g-i, 200 μg mL^{-1}) j: *β*-glucosidase (4 U mL^{-1}), 10% DMSO-mixed PBS, pH 7.4].

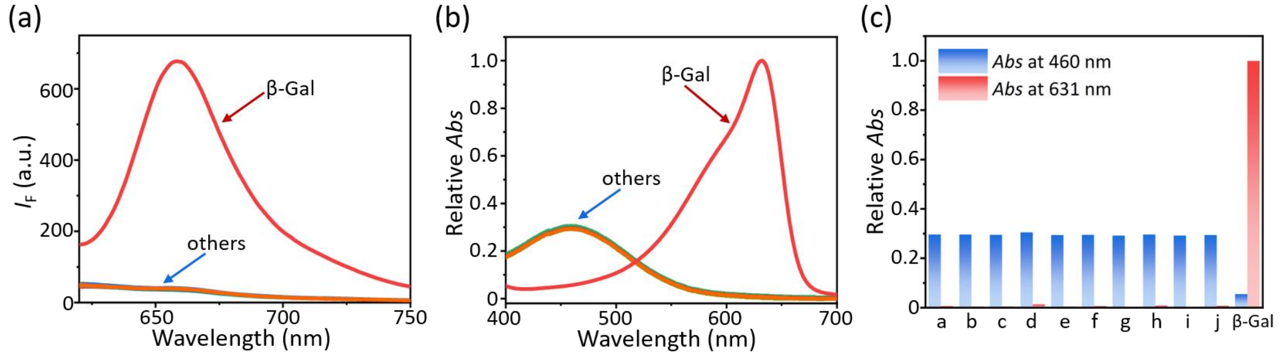

Figure S9. (a) Fluorescence spectra of **TF2** incubated with *β*-Gal (4 U mL^{-1}) and other analytes for 1 h (λ_{ex} = 570 nm, T = 37°C, 10% DMSO-mixed PBS, pH 7.4). (b) Relative absorption spectra and (c) corresponding absorption intensity comparison at 460 nm and 631 nm of **TF2** with *β*-Gal (4 U mL^{-1}) and other analytes for 1 h [a: blank, b: H_2O_2, c: NaClO, d: Hcy, e: Cys, f: GSH, (a-f, 200 μM) g: DNA, h: BSA, i: cellose (g-i, 200 μg mL^{-1}) j: *β*-glucosidase (4 U mL^{-1}), 10% DMSO-mixed PBS, pH 7.4].

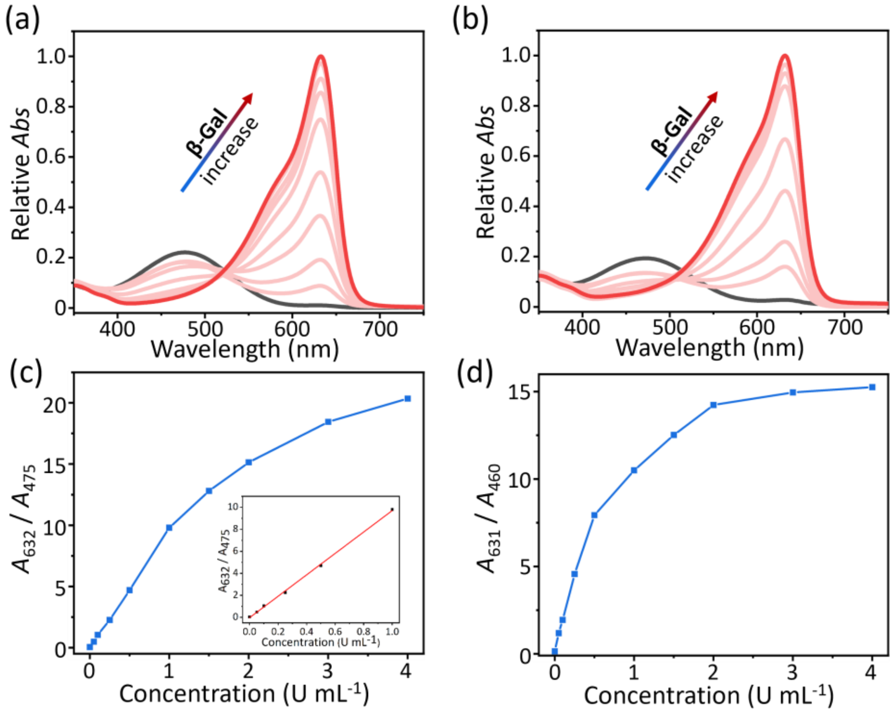

Figure S10. Ratiometric absorption spectra of (a) **TF1** and (b) **TF2** (10 μM) incubated with different concentration of *β*-Gal (0-4 U mL^{-1}). Plotting the (c) A_{632} / A_{475} of **TF1** and (d) the A_{631} / A_{460} **TF2** (10 μM) incubated with different concentration of *β*-Gal (0-4 U mL^{-1}). Inset: fitted A_{632} / A_{475} curve of **TF1** incubated with low concentration of *β*-Gal (0-0.5 U mL^{-1}). All absorption spectra were measured after probes incubated with *β*-Gal at 37°C in PBS (pH 7.4) mixed with 10% DMSO.

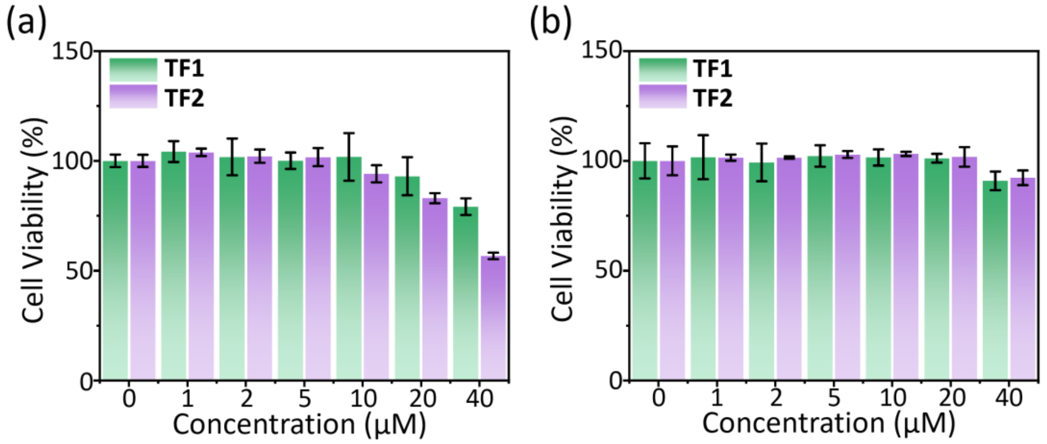

Figure S11. Cell viability of (a) normal and (b) senescent A549 cells after incubation with different concentrations (0-40 μM) of **TF1** (green) and **TF2** (purple) for 24 h. S. D. means standard deviation (n = 3).

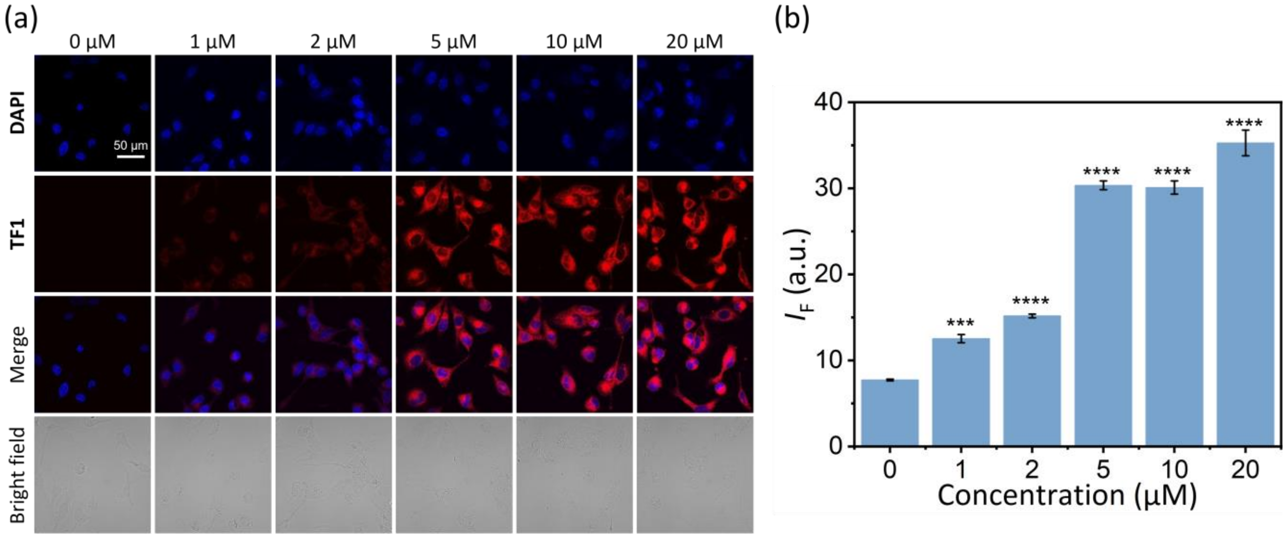

Figure S12. (a) Fluorescence imaging and (b) quantitative signal intensity of senescent A549 cells after incubating with different concentrations (0-20 μM) of **TF1** for 4 h. Excitation and emission wavelength of **TF1** were 640 nm and 650–700 nm, respectively. Error bars represent the S.D. ***$p < 0.001$, ****$p < 0.0001$; n = 3.

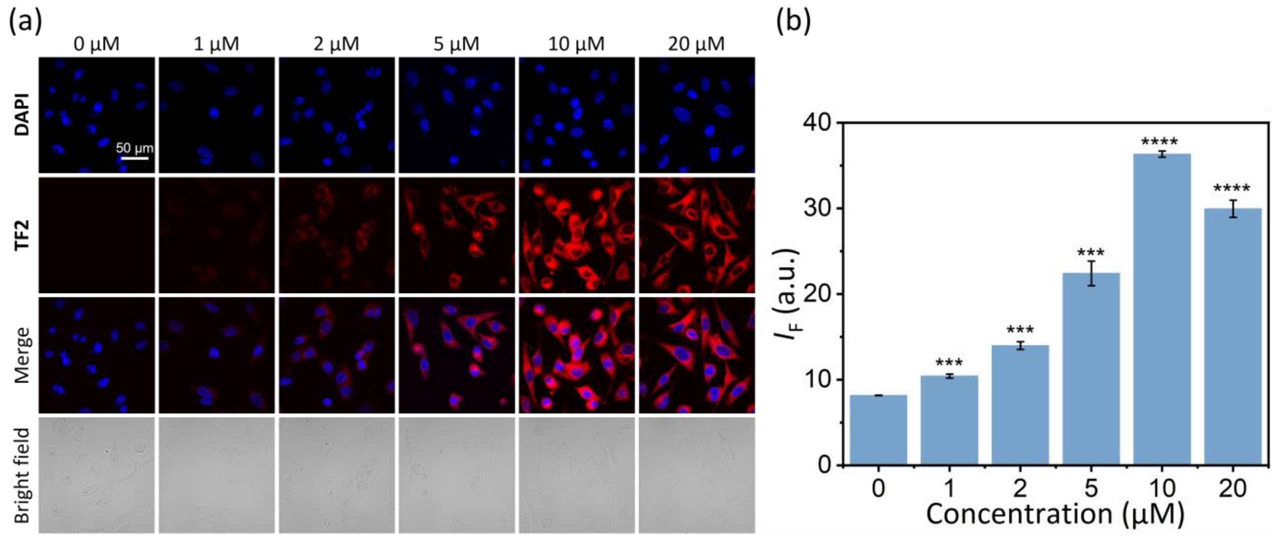

Figure S13. (a) Fluorescence imaging and (b) quantitative signal intensity of senescent A549 cells after incubating with different concentrations (0–20 μM) of **TF2** for 4 h. Excitation and emission wavelength of **TF2** were 640 nm and 650–700 nm, respectively. Error bars represent the S.D. ***p < 0.001, ****p < 0.0001; n = 3.

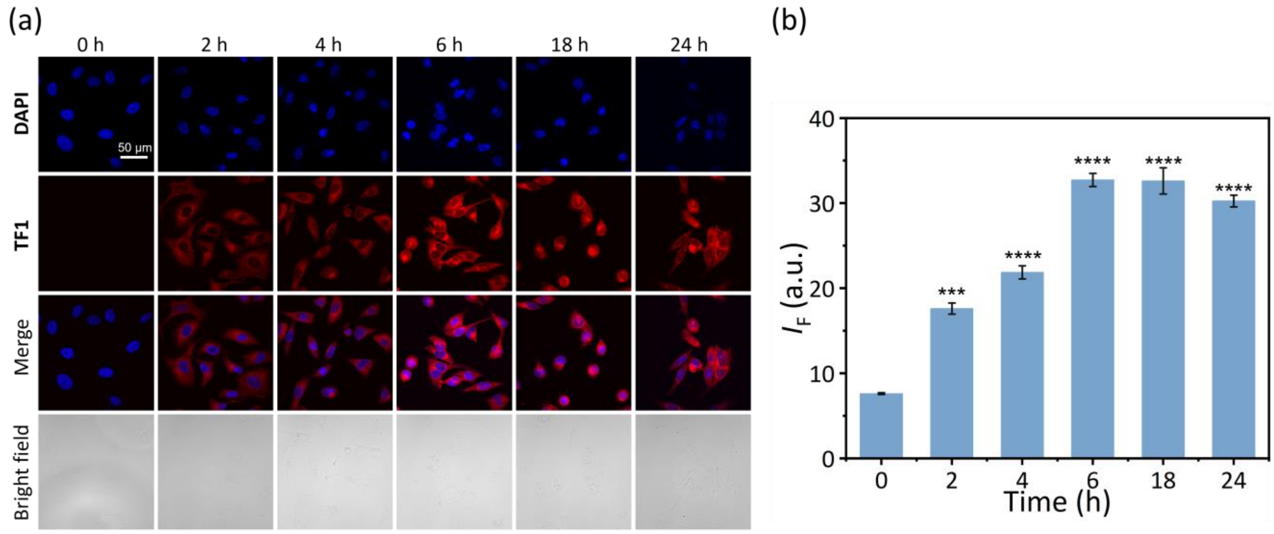

Figure S14. (a) Fluorescence imaging and (b) quantitative signal intensity of senescent A549 cells after incubating with **TF1** (10 μM) for different times (0–24 h). Excitation and emission wavelength of **TF1** were 640 nm and 650–700 nm, respectively. Error bars represent the S.D. ***p < 0.001, ****p < 0.0001; n = 3.

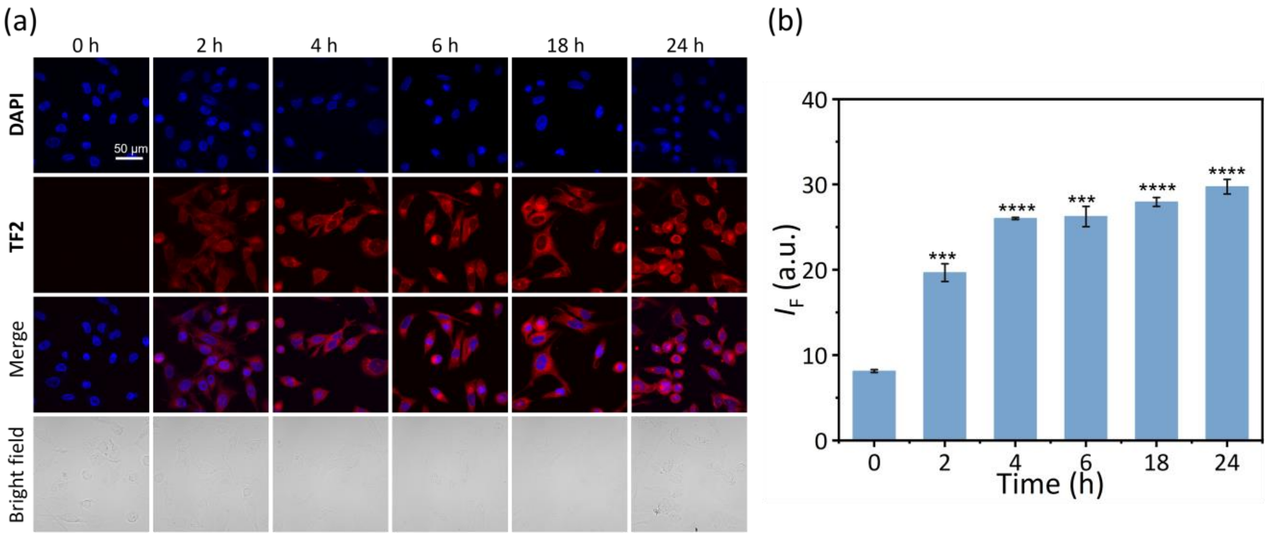

Figure S15. (a) Fluorescence imaging and (b) quantitative signal intensity of senescent A549 cells after incubating with **TF2** (10 μM) for different times (0–24 h). Excitation and emission wavelength of **TF2** were 640 nm and 650-700 nm, respectively. Error bars represent the S.D. ***p < 0.001, ****p < 0.0001; n = 3.

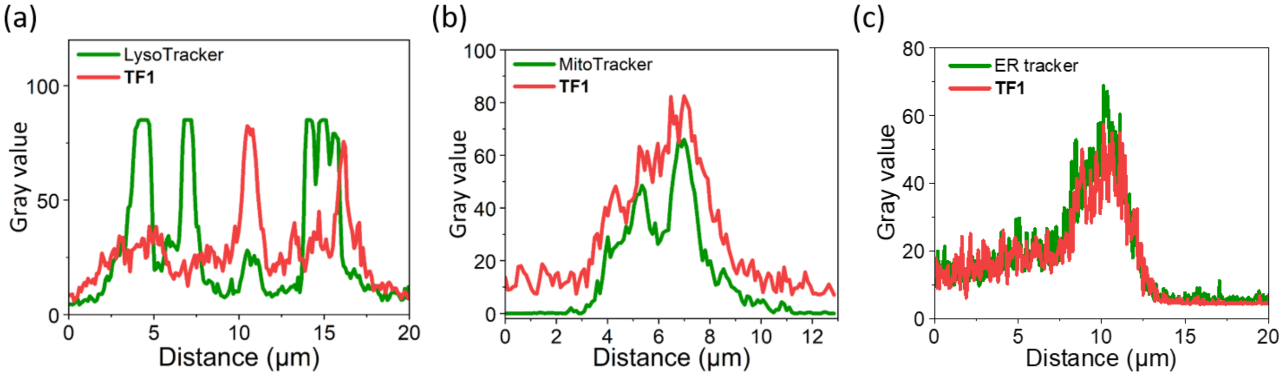

Figure S16. Fluorescence co-localization of **TF1** (10 μM) with (a) LysoTracker (a lysosome tracker, 75 nM), (b) MitoTracker (a mitochondria tracker, 75 nM) and (c) ERTracker (an endoplasmic reticulum tracker, 75 nM).

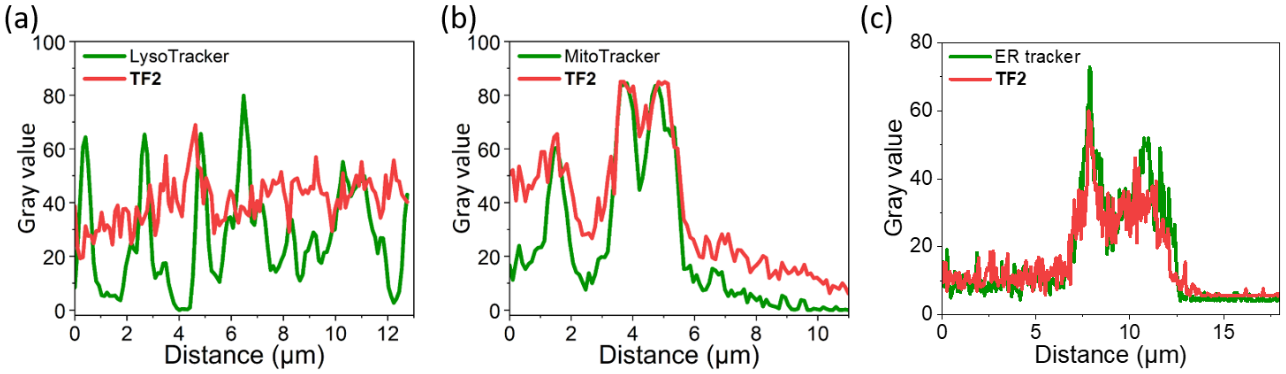

Figure S17. Fluorescence co-localization of **TF2** (10 μM) with (a) LysoTracker (a lysosome tracker, 75 nM), (b) MitoTracker (a mitochondria tracker, 75 nM) and (c) ERTracker (an endoplasmic reticulum tracker, 75 nM).

^{1}H NMR and ^{13} NMR Spectra

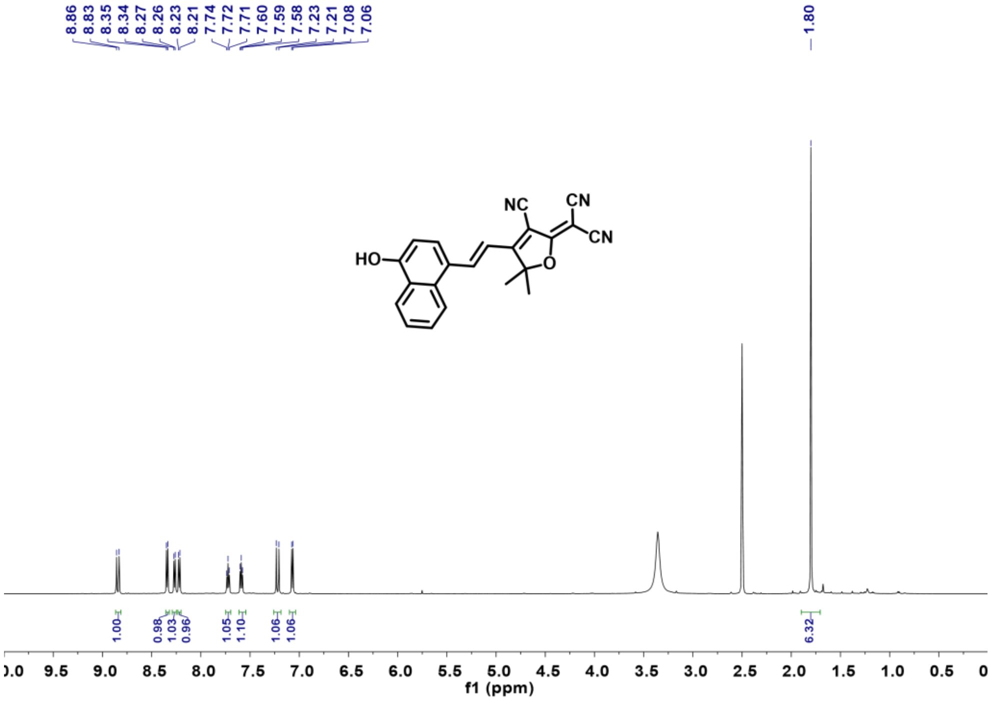

Figure S18. ^{1}H-NMR of compound **TF1-OH** in DMSO-d_6.

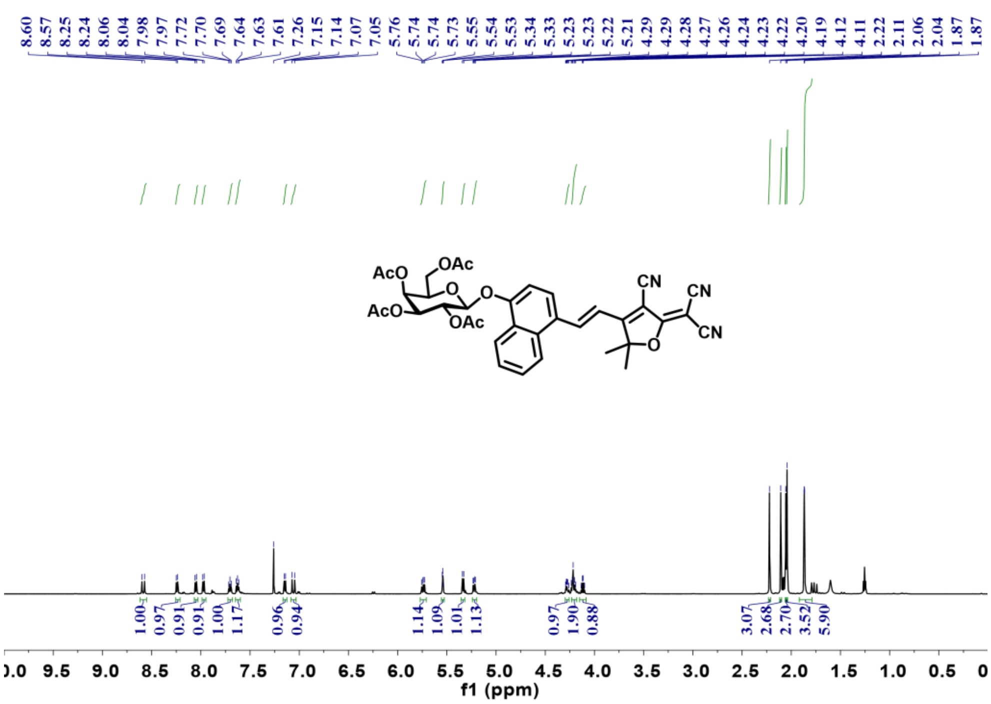

Figure S19. ^{1}H-NMR of compound **TF1-GalAc** in $CDCl_3$.

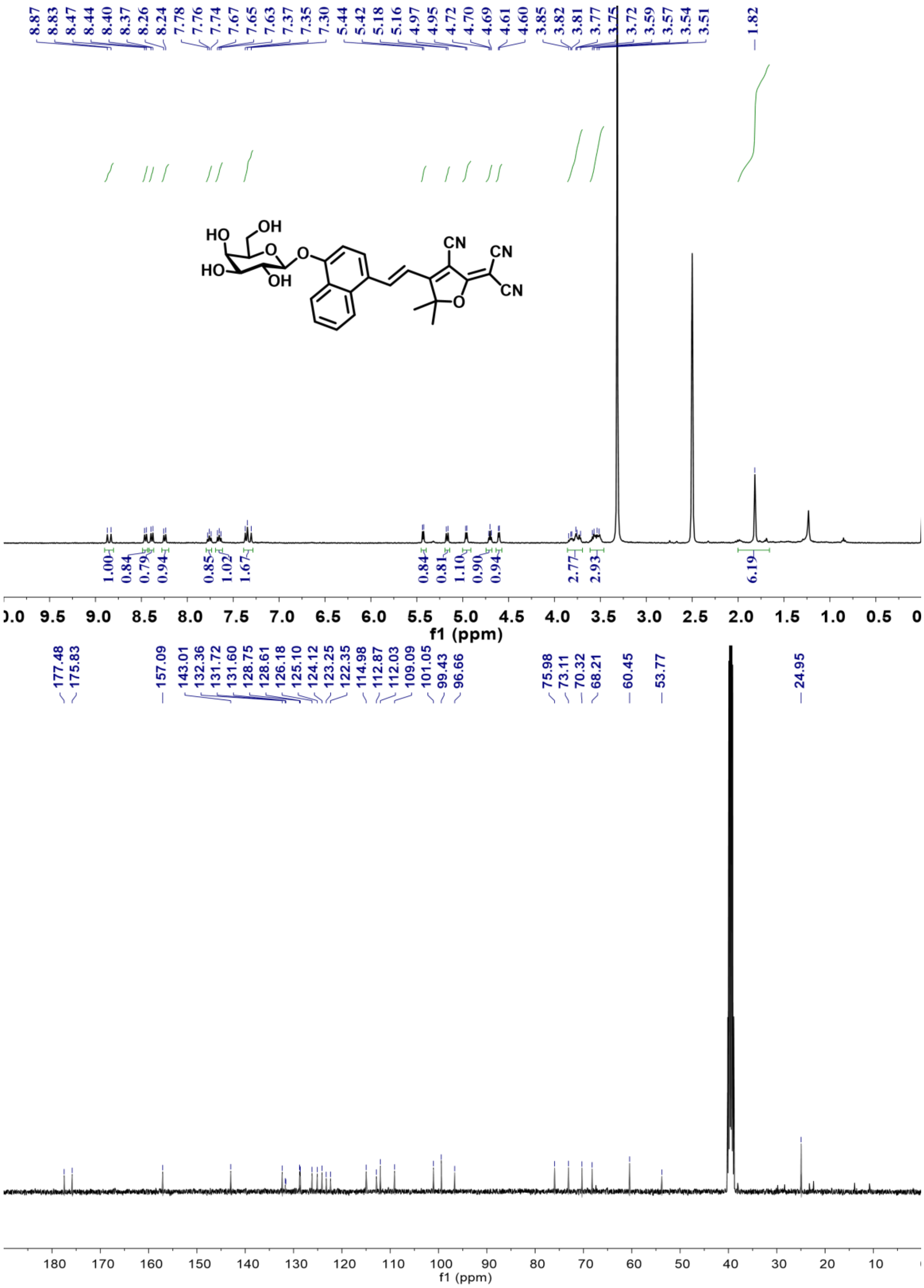

Figure S20. ^{1}H-NMR and ^{13}C-NMR of compound **TF1** in DMSO-d_6.

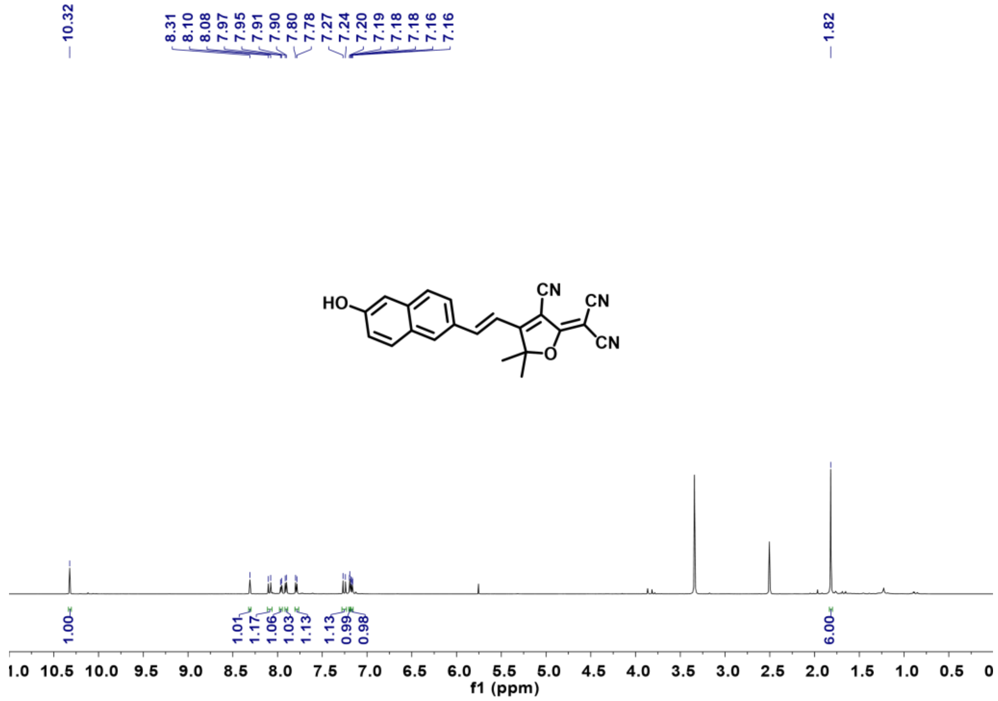

Figure S21. ^{1}H-NMR of compound **TF2-OH** in DMSO-d_6.

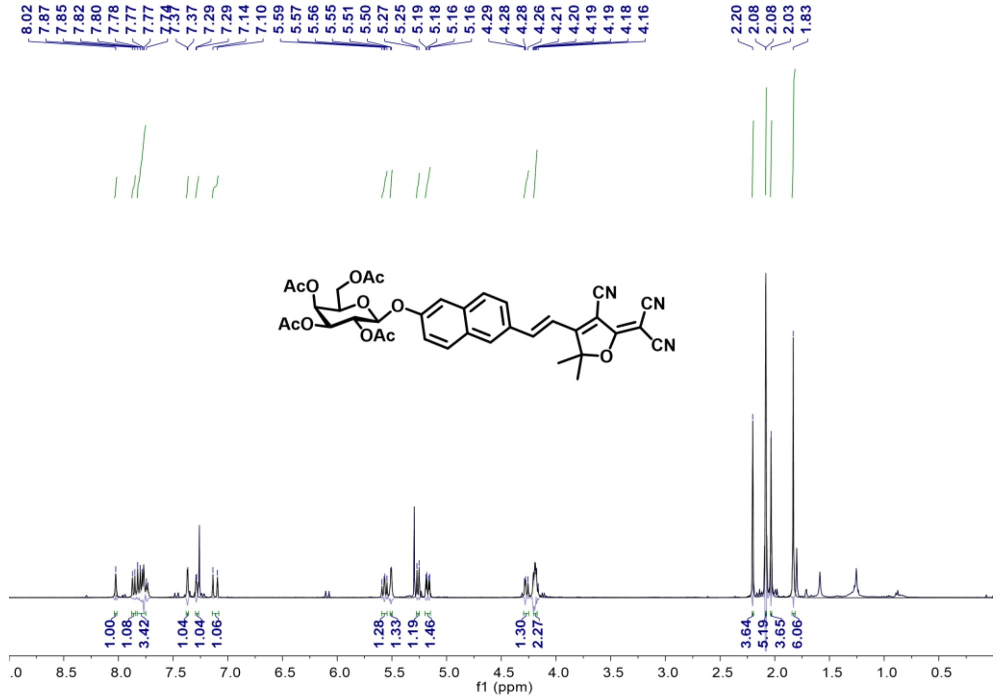

Figure S22. ^{1}H-NMR of compound **TF2-GalAc** in $CDCl_3$.

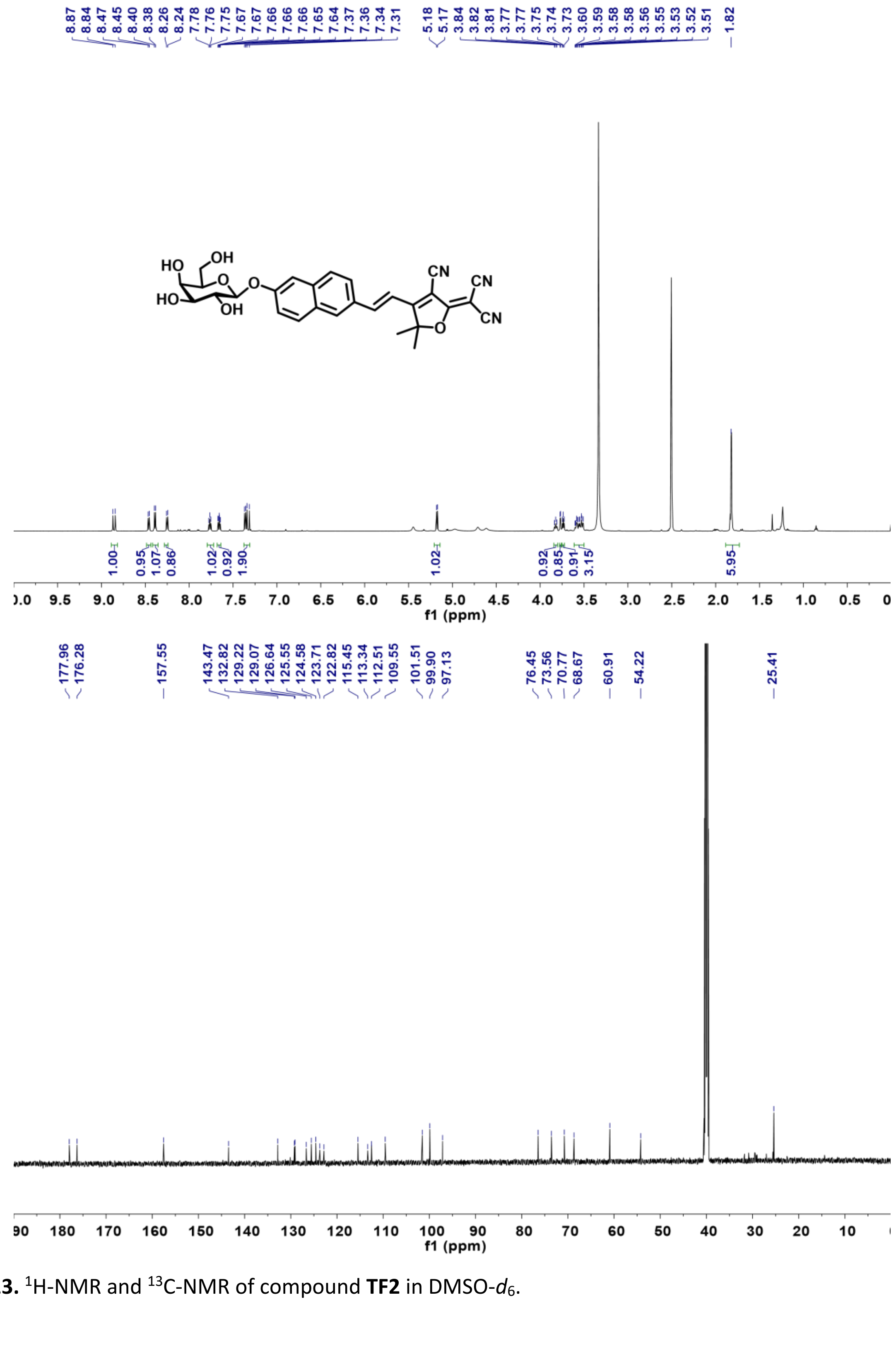

Figure S23. ^{1}H-NMR and ^{13}C-NMR of compound **TF2** in DMSO-d_6.

Elemental Composition Report **Page 1**

Single Mass Analysis
Tolerance = 5.0 mDa / DBE: min = -1.5, max = 50.0
Element prediction: Off
Number of isotope peaks used for i-FIT = 3

Monoisotopic Mass, Even Electron Ions
1175 formula(e) evaluated with 1 results within limits (up to 50 best isotopic matches for each mass)
Elements Used:
C: 28-28 H: 25-25 N: 0-100 O: 0-100 Na: 0-1
2
240930-5-HY-88 31 (0.219)

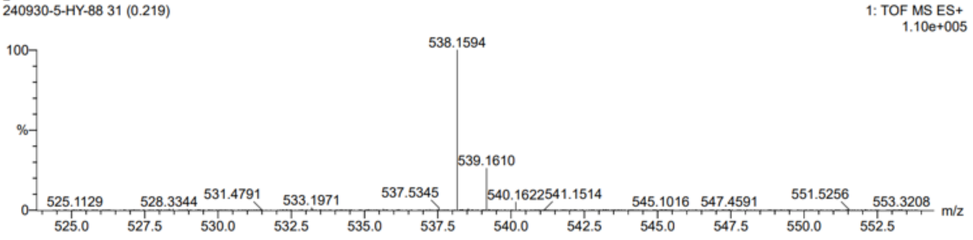

Minimum:				-1.5				
Maximum:		5.0	10.0	50.0				
Mass	Calc. Mass	mDa	PPM	DBE	i-FIT	Norm	Conf(%)	Formula
538.1594	538.1590	0.4	0.7	17.5	493.9	n/a	n/a	C28 H25 N3 O7 Na

Figure S24. HRMS spectral of **TF1**.

Elemental Composition Report **Page 1**

Single Mass Analysis
Tolerance = 5.0 mDa / DBE: min = -1.5, max = 50.0
Element prediction: Off
Number of isotope peaks used for i-FIT = 3

Monoisotopic Mass, Even Electron Ions
1175 formula(e) evaluated with 1 results within limits (up to 50 best isotopic matches for each mass)
Elements Used:
C: 28-28 H: 25-25 N: 0-100 O: 0-100 Na: 0-1
2
240930-5-HY-89 35 (0.240)

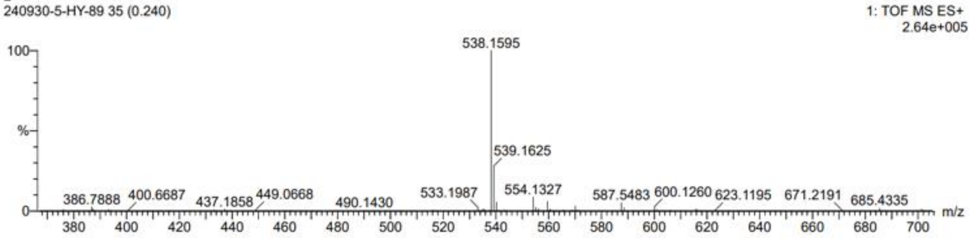

Minimum:				-1.5				
Maximum:		5.0	10.0	50.0				
Mass	Calc. Mass	mDa	PPM	DBE	i-FIT	Norm	Conf(%)	Formula
538.1595	538.1590	0.5	0.9	17.5	624.8	n/a	n/a	C28 H25 N3 O7 Na

Figure S25. HRMS spectral of **TF2**.

References

[1] Yan, K.-C.; Thet, N.; Heylen, R. A.; Sedgwick, A. C.; James, T. D.; Jenkins, A. T. A. and He, X.-P., Repurposing a long-wavelength fluorescent boronate probe for the detection of reactive oxygen species (ROS) in bacteria, *Sens. Diagn.* **2023**, *2*, 1181-1185.

[2] Gaussian 16, R. B., M. J. Frisch, G. W. Trucks, H. B. Schlegel, G. E. Scuseria, M. A. Robb, J. R. Cheeseman, G. Scalmani, V. Barone, G. A. Petersson, H. Nakatsuji, X. Li, M. Caricato, A. V. Marenich, J. Bloino, B. G. Janesko, R. Gomperts, B. Mennucci, H. P. Hratchian, J. V. Ortiz, A. F. Izmaylov, J. L. Sonnenberg, D. Williams-Young, F. Ding, F. Lipparini, F. Egidi, J. Goings, B. Peng, A. Petrone, T. Henderson, D. Ranasinghe, V. G. Zakrzewski, J. Gao, N. Rega, G. Zheng, W. Liang, M. Hada, M. Ehara, K. Toyota, R. Fukuda, J. Hasegawa, M. Ishida, T. Nakajima, Y. Honda, O. Kitao, H. Nakai, T. Vreven, K. Throssell, J. A. Montgomery, Jr., J. E. Peralta, F. Ogliaro, M. J. Bearpark, J. J. Heyd, E. N. Brothers, K. N. Kudin, V. N. Staroverov, T. A. Keith, R. Kobayashi, J. Normand, K. Raghavachari, A. P. Rendell, J. C. Burant, S. S. Iyengar, J. Tomasi, M. Cossi, J. M. Millam, M. Klene, C. Adamo, R. Cammi, J. W. Ochterski, R. L. Martin, K. Morokuma, O. Farkas, J. B. Foresman, and D. J. Fox, Gaussian, Inc., Wallingford CT, 2016.

[3] Lu, T. and Chen, F., Multiwfn: A multifunctional wavefunction analyzer, *J. Comput. Chem.* **2012**, *33*, 580-592.

[4] Liu, Z.; Lu, T. and Chen, Q., An sp-hybridized all-carboatomic ring, cyclo[18]carbon: Electronic structure, electronic spectrum, and optical nonlinearity, *Carbon* **2020**, *165*, 461-467.

[5] Lu, T., A comprehensive electron wavefunction analysis toolbox for chemists, Multiwfn, *J. Chem. Phys.* **2024**, *161,* 802503.
